# Mental Health, Gender Dysphoria, and Quality of Life in Trans Individuals Following Top Surgery: Protocol for a Prospective Cohort Study

**DOI:** 10.2196/83928

**Published:** 2026-08-21

**Authors:** Julia Bak, Kah Aik Tan, Jack Ball, Sam Bonney, Julia K Moore, Kirsty Hird, Blake S Cavve, Larissa Marion, Tim Hewitt, Sav Zwickl, Simone Mahfouda, Ashleigh Lin, Penelope Strauss

**Affiliations:** 1 Youth Mental Health, The Kids Research Institute Australia, 15 Hospital Ave, Nedlands WA 6009, Perth, Australia, 61 63191000; 2 South Metropolitan Health Service - Mental Health, Fiona Stanley Hospitals Group, Perth, Australia; 3 East Metropolitan Health Service - Mental Health, Royal Perth Bentley Hospitals Group, Perth, Australia; 4 The Gender Diversity Service, Child and Adolescent Health Service – Mental Health, Perth Children's Hospital, Perth, Australia; 5 The Kids Research Institute Australia, 15 Hospital Ave, Nedlands WA 6009, Perth, Australia; 6 School of Psychological Science, The University of Western Australia, Perth, Australia; 7 Fellow of the Royal Australasian College of Surgeons, Perth, Australia; 8 Child and Adolescent Health Service, Perth Children's Hospital, Perth, Australia; 9 Department of Medicine (Austin Health), Trans Health Research Group, The University of Melbourne, Perth, Australia; 10 Department of Clinical Psychology, Acute Adult Inpatient Services, Graylands Hospital, Perth, Australia; 11 School of Population and Global Health, The University of Western Australia, Perth, Australia; 12 Australian Research Centre in Sex, Health and Society, La Trobe University, Melbourne, Australia

**Keywords:** transgender, gender dysphoria, top surgery, chest reconstruction, gender affirmation

## Abstract

**Background:**

Top surgery refers to various gender-affirming surgical procedures involving breast or chest tissue. Existing research suggests top surgery improves physical, psychological, and social well-being in transgender and gender-diverse individuals (herein “trans”) who desire it. However, most studies have been retrospective, have not used validated outcome measures, and have excluded nonbinary individuals, limiting the robustness of the existing research.

**Objective:**

This study aims to examine changes in mental health, gender dysphoria, risky behaviors, and quality of life of trans people (including nonbinary individuals) following top surgery.

**Methods:**

Trans individuals aged 16 years and older undergoing top surgery will be recruited from a single surgeon’s office in Perth, Western Australia, as part of a longitudinal, prospective cohort study. Questionnaires will be administered to participants at 3 time points: within 8 weeks prior to surgery, 6‐8 weeks post surgery, and 1 year post surgery. These questionnaires will collect data on patient demographics, planning and expectations for surgery, support received during surgery access, satisfaction with care received from their surgeon, use of gender-affirming hormones, mental health (ie, anxiety, depression, self-harm and suicide, and mental health diagnoses), gender euphoria and dysphoria, exercise and diet, disordered eating, quality of life, substance use, social acceptance and discrimination, and reflections on the experience and outcomes of their surgery. Patient-reported outcomes will be measured using validated instruments and bespoke items developed through community consultation. Validated instruments include the GENDER-Q (health professional subscale); the Generalized Anxiety Disorder 7-item; the Patient Health Questionnaire-9; the Gender Euphoria Scale; an adapted version of the Olson-Kennedy Chest Dysphoria Scale; the International Physical Activity Questionnaire - Short Form; the Eating Disorder Examination Questionnaire - Short; the Assessment of Quality of Life 8 Dimensions; and the Alcohol, Smoking, and Substance Involvement Screening Test - Short Form. Surgical satisfaction will be assessed using both patient- and surgeon-reported measures, enabling between-group comparisons. Quantitative data will be analyzed using linear regression models and repeated-measures ANOVA to compare outcomes across the 3 time points; qualitative data will undergo a general inductive approach to analysis to produce categories that contextualize and supplement the quantitative findings, as well as identify any novel or unexpected experiences.

**Results:**

This study received funding in February 2024. Data collection commenced in May 2025 and is expected to be completed by December 2027. As of February 2026, 26 people have consented to participate.

**Conclusions:**

This study will extend research on the mental health, gender dysphoria, and quality-of-life outcomes of top surgery for trans individuals in Australia. By addressing limitations of existing research, this study aims to improve our understanding of trans people’s experience accessing and receiving top surgery. The findings may guide improvements in perioperative and postoperative care and support for patients and inform policy related to gender-affirming surgeries in Australia.

## Introduction

### Background

Population estimates suggest that transgender and gender diverse people (herein “trans”) comprise 7.1% (486/6841) of Australia’s high school-aged population [1], 1.1% (22/2000) of adults aged 25‐44 years, and 0.5% (14/3003) of those aged 45 years and older [2]. These figures align with international estimates, which suggest that 0.5% to 4.5% of adults worldwide are trans [3]. Trans people are known to experience poorer mental health than their cisgender peers [4-6]. In the largest Australian survey of trans young people to date, 3 in 4 had ever been diagnosed with depression or anxiety and almost 1 in 2 had ever attempted suicide [5]. Comparable rates of depression (663/914, 73%) have been reported in trans adults, while 67% (613/914) have been diagnosed with anxiety and 43% (393/914) have attempted suicide [7]. When compared to the general adult population in Australia [8], self-reported rates of suicide attempts are 9 times higher in trans adults [9] and 10 times higher in trans young people [5].

Many trans people experience gender dysphoria [10], which refers to distress that arises from an incongruence between one’s experienced gender and one’s gender presumed at birth [11]. Gender dysphoria can be related to specific bodily characteristics (eg, the chest, hips, and voice), cultural expectations of gender, how others perceive a person’s gender (eg, “passing” as one’s affirmed gender), how a person is affirmed or not affirmed by others (eg, being gendered correctly or experiencing misgendering), or any combination of the aforementioned [12]. The onset or exacerbation of gender dysphoria is often associated with changes during puberty and the development of secondary sex characteristics [12], though both the *DSM-5-TR* (*Diagnostic and Statistical Manual of Mental Disorders, Fifth Edition, Text Revision*) [11] and the *ICD-11* (*International Statistical Classification of Diseases and Related Health Problems, Eleventh Revision*) [13] state that gender dysphoria can also be diagnosed in earlier childhood. For instance, many trans people report their earliest experience of gender dysphoria as occurring between the ages of 3 and 7 years [14,15]. Gender dysphoria is associated with a number of negative mental health outcomes such as depression [16-19], anxiety [16-19], suicidal thoughts and behaviors [20,21], and poor quality of life [19].

In addition, gender dysphoria is associated with a number of risky behaviors, including nonsuicidal self-injury [17,21,22], disordered eating [23,24], compulsive exercise [25,26], low levels of physical activity [25], and substance use [27,28]. Behaviors such as nonsuicidal self-injury, disordered eating, and substance use are commonly used as coping strategies [29-32] and, as such, may be common among trans people seeking to manage the distress associated with gender dysphoria. However, many of these behaviors may also be used to alter one’s appearance in an attempt to meet the sociocultural appearance ideals of one’s gender. For example, some trans people report restricting their calorie intake or avoiding physical activity in order to be thin and avoid looking muscular, or to suppress curves [23,33], whereas others may engage in compulsive exercise to try to increase weight and muscle mass [34,35]. Additionally, some trans people assigned female at birth may avoid physical exercise that may trigger dysphoria by bringing awareness to the breasts (eg, running and swimming) [36]. Each of these risky behaviors can yield a number of detrimental health outcomes [25,37-40].

For many trans people who experience gender dysphoria, gender-affirming health care offers various approaches to alleviate this distress and any subsequent negative outcomes. Gender-affirming health care refers to social, behavioral, psychological, and medical interventions that support and respect an individual’s gender identity [14,15]. Medical gender-affirming health care, which includes gender-affirming surgeries, comprises interventions that help alleviate gender dysphoria in many trans people [41]. However, it is important to recognize that not all trans people pursue medical interventions, nor are all trans people who desire such interventions able to access them [9].

Gender-affirming surgeries can include “bottom” surgeries (eg, phalloplasty and vaginoplasty), facial surgeries (also known as “facial feminization” or “facial masculinization” surgeries), vocal surgery, and “top” surgeries [42]. The phrase “top surgery” is used within this study as an umbrella term for all gender-affirming chest and breast surgeries, including both chest reconstructive procedures and breast augmentation. Chest reconstruction and breast reduction involve the removal of breast tissue, sometimes with additional skin removal [43]. Breast augmentation often involves inframammary incisions to insert implants, the size of which depends on a patient’s preference and chest anatomy [43,44]. Both chest reconstructive surgeries and breast augmentation may also involve the repositioning and resizing of the nipple areolar complex [42], and chest reconstruction may include contouring to create a more masculine or gender-affirming chest shape [45]. One recent meta-analysis suggests top surgeries are the most common gender-affirming surgeries accessed by trans people [46], with 56.6% of trans people who had accessed gender-affirming surgeries having had either chest or breast procedures, compared to 35.1% having had bottom surgeries and 13.9% having had other facial or cosmetic procedures. Some studies report top surgeries as being up to twice as common as bottom surgeries [7,47,48].

Top surgery has been associated with improved physical, psychological, and social well-being, including significant improvements in quality of life [44,49-51] and improved mental health and behavioral outcomes [44,49-55]. For instance, top surgery has been associated with reductions in depression, anxiety, and suicidality, both when comparing trans people before and after surgery [51-55] and when comparing those who have and have not undergone the procedure [54]. It has also been associated with decreased gender dysphoria and dysphoria related to the chest or breasts, also known as chest dysphoria [51,52,55], as well as lowered rates of eating disorders [56]. Both adolescent and adult trans populations have reported high levels of satisfaction following top surgeries [51,52,55], with low complications and rates of regret [51,52,55]. In a meta-analysis of regret following top surgery in trans adolescents and adults (aged 13 years and older), the pooled prevalence of regret was reported as 1% [57]. Among individuals undergoing feminizing top surgeries, regret ranged from less than 1% to 2%, while for those undergoing masculinizing surgeries, regret was less than 1%; this is consistent with previous pooled estimates showing regret rates between 1% and 1.5% [58].

Nonsurgical gender-affirming interventions—such as chest binding or breast forms—are important, reversible alternatives for those unable to, or not wishing to, access top surgeries. Though temporary, these options have been shown to reduce gender dysphoria and improve mental health and quality of life [59,60]. However, nonsurgical alternatives also have important limitations. For example, extended or improper chest binding can pose significant physical health risks, including back pain, shortness of breath, chest pain, and skin irritation [61,62], and both binding and breast forms require daily application, offering only temporary relief from chest dysphoria. For these reasons, many trans people ultimately prefer and pursue top surgery for its ability to provide permanent, comprehensive management of chest dysphoria [36,63].

Although current evidence supports the use of top surgery in the management of gender dysphoria, there are several methodological limitations in the existing research. Most studies are retrospective [51,64-67], have follow-up periods of less than 1 year [68,69], do not control for concurrent gender-affirming treatments [57,70,71], and do not specifically evaluate mental health and quality-of-life outcomes following surgery [53,72,73]. Retrospective designs can be subject to bias, as participants are expected to recall presurgical functioning, while short follow-up periods cannot establish whether improvements persist or how outcomes evolve over time. The few studies with longer-term follow-up periods are retrospective with high attrition rates, introducing survivorship bias that may inflate positive outcomes and limit generalizability. While the limited prospective research demonstrates high postsurgical satisfaction (90%‐93%) [49,52] and improved mental health outcomes [52,55,74,75], these studies are constrained by small sample sizes [63,76], follow-up periods of 6 months or less [49,51,52,74], and lack of comparison or control groups [49,52,75-77]. Prospective designs with longer follow-up periods are therefore needed to accurately measure changes from baseline, identify how improvements change over time, and examine whether outcomes are sustained over the long term.

Additionally, most studies originate from the United States and Europe [51,55,65-67,78], limiting external validity for other cultures and health care systems in other countries. Different national contexts present distinct financial, cultural, and societal landscapes that influence not only access to top surgery, but also the quality and continuity of peri- and postoperative care, availability of social support, and exposure to stigma and discrimination—all factors that can shape health outcomes [79-81]. In the United States, structural stigma—including specific anti-transgender legislation and hostile political climates—has been associated with poorer mental health among trans people, independent of direct barriers to care [79,80]. While the United Kingdom offers publicly funded top surgery, long wait times have been associated with poorer mental health during the waiting period [81]. In Australia, waitlists are typically shorter; however, comprehensive public funding for top surgery is lacking, with most procedures occurring in the private sector and out-of-pocket costs ranging from AUD $4000 to AUD $18,000 (US $2844 to US $12,797) [82,83]. Collectively, these findings suggest that each country’s unique health care, legislative, and social contexts may differentially influence health outcomes following top surgery. Australian-specific research is therefore needed to understand how these contextual factors operate locally and to inform evidence-based clinical practice and policy.

An additional limitation of the current literature is the scarcity of validated tools to measure health outcomes following top surgery among trans people specifically [55,84,85]. Most studies have used measures designed for cisgender people [44,49,55,84,85], such as the BREAST-Q [86], or tools developed to assess disordered eating, such as the Body Uneasiness Test [87]. Other studies have measured health outcomes with unvalidated Likert scales [55,84,86,88,89]. However, these tools do not capture the specific experiences of trans people (such as distress arising from gender dysphoria; the unique, positive impact of gender euphoria; or the functional limitations associated with chest binding), which limits their utility in studies on gender-affirming procedures. Recent research has seen the development and early validation of trans-specific measures that show promise for future work in this space. The Olson-Kennedy Scale [36], a validated measure of chest dysphoria, addresses this gap by assessing some of the experiences central to trans people, including binding behaviors, avoidance of gendered spaces, and limitations on social participation and sexual relationships. In addition, the Gender Euphoria Scale (GES) is a novel tool designed to assess positive feelings about one’s gender [90], and the GENDER-Q [91], adapted from the BREAST-Q and the BODY-Q [92,93], is a newly developed patient-reported measure of gender-affirming care outcomes. Unlike existing instruments, the GENDER-Q also explicitly included nonbinary individuals in its validation samples, demonstrating the measure’s applicability across diverse gender identities and gender-affirming trajectories. Taken together, these developments highlight the importance of using instruments developed and validated for use with trans samples to ensure postsurgical outcomes are measured accurately and meaningfully.

Few studies on top surgery to date have included nonbinary participants [64,74,94]. This could be attributed to the misconception that nonbinary people do not wish to pursue medical gender-affirming care [95]. While nonbinary people access medical gender affirmation at lower rates than binary trans people [96,97], Australian estimates suggest that 25.1% of nonbinary people have had at least one gender-affirming surgery and 23.6% are taking steps to access gender-affirming surgeries [48]. In addition, studies suggest nonbinary people experience distinct barriers when attempting to access medical gender-affirming care, including pressure to transition to a binary gender and a lack of information on gender-affirming care specific to nonbinary gender identities [98]. Nonbinary people seeking chest masculinization procedures have also been shown to differ from binary trans people in their aesthetic preferences [94,99]. However, a recent systematic review found no studies discussing surgical techniques or outcomes in nonbinary patients undergoing chest feminization [94]. Despite these findings suggesting that nonbinary individuals have distinct surgical preferences and experiences, there have been limited studies on outcomes specific to nonbinary individuals undergoing top surgery [74]. This underscores the need to include nonbinary individuals when evaluating the effects of top surgery on the mental health and quality of life of trans people [74,100,101].

### Current Research

Top surgery is anticipated to improve mental health and quality of life in trans individuals and reduce the distress associated with gender dysphoria. However, few studies have specifically examined detailed mental health and behavioral outcomes following top surgery [53,72,73], and of those that have, fewer used validated instruments [84]. In addition, most studies are retrospective in design [64,66,84], have short follow-up periods [64,66,68,69], and do not account for nonbinary participants [64,68,69,74,94]. Thus, this prospective cohort study aims to fill these knowledge gaps by longitudinally evaluating the effects of top surgery on the mental health, risky behaviors, gender dysphoria, and quality of life of trans people, including nonbinary people.

We hypothesize that there will be reductions in negative mental health outcomes (ie, gender dysphoria, depression, anxiety, and suicidal thoughts and behaviors) and risky behaviors (self-harm, substance use, disordered eating, compulsive exercise, and avoidance of physical activity), and improvements in positive outcomes (ie, gender euphoria and quality of life) at 6‐8 weeks post surgery relative to baseline scores, and that these changes will be maintained at 1 year post surgery. Additionally, we hypothesize that individuals who report higher satisfaction with surgical outcomes will experience greater reductions in negative outcomes and greater improvements in positive outcomes compared to those with lower self-reported satisfaction.

## Methods

### Study Design

**Figure 1.**
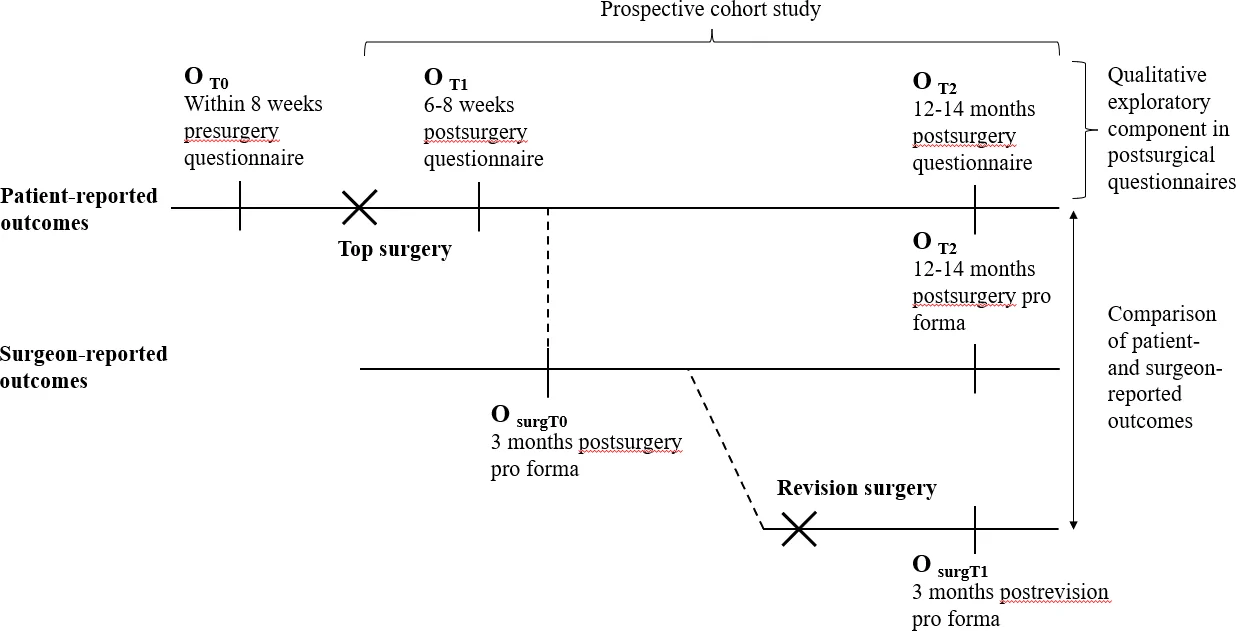
Schematic overview of the study design.

We are currently conducting a prospective cohort study of trans people aged 16 years and older undergoing top surgery in Perth, Western Australia. Participants complete measures over 3 time points: within 8 weeks prior to surgery (T0), 6‐8 weeks post surgery (T1), and 1 year post surgery (T2). If a participant’s surgery is postponed by 4 or more weeks and a participant has already completed their presurgery questionnaire, they are asked to complete the presurgery questionnaire again within 8 weeks of their new surgery date. The previous presurgery questionnaire data are then disregarded. Outcomes measured include mental health, gender dysphoria, gender euphoria, and quality of life. Bespoke questions developed through community consultation are also included. Experiences accessing surgical care, including barriers and available supports, are measured at T0, while follow-up questionnaires investigate recovery from top surgery, including support received and surgical satisfaction (T1 and T2). With consent from the participant, the operating surgeon’s satisfaction with surgical outcomes is reported in a pro forma 3 months after surgery (surgT0), coinciding with the patient’s 3-month follow-up appointment. If revision surgeries are required, the operating surgeon completes additional pro formas for each added surgery (surgT1; additional time points to be added as needed). An overview of the study design is provided in Figure 1.

### Ethical Considerations

This study received ethics approval from the University of Western Australia Human Research Ethics Committee (2021/ET000069) in accordance with the National Statement on Ethical Conduct in Human Research (2023). Written informed consent is obtained from all participants prior to data collection via an online consent form administered through Qualtrics. For participants aged 16 to 18 years, the patient and their parent or guardian are invited to attend a videoconference or telephone call to discuss the study with the research team. If the young person is interested in participating, they and their parent or guardian are each sent a link to complete their respective consent forms online. No compensation is provided to participants for their involvement in this study.

Participant data are deidentified, with names and identifying information replaced with unique study codes. Staff from the surgeon’s office pass contact information to the research team, but the participating surgeon does not know which patients are participating until after surgery when they are asked to complete the surgeon pro forma. The surgeon does not have access to participants’ self-report questionnaire data at any stage. All data are stored on password-protected, encrypted servers accessible only to members of the research team.

### Recruitment and Consent

#### Eligibility and Setting

Eligible participants are trans people aged 16 years or older who have consented to top surgery, have not had top surgery previously (ie, are not undertaking a revision surgery), and have sufficient English comprehension to consent and complete assessments. Participants will be recruited from a single surgeon’s office in Perth, Western Australia, with the intention of expanding to additional sites in the future. The participating surgeon does not currently perform surgeries on patients younger than the age of 18 years, and hence all participants will be at least 17 years when completing the questionnaire at T0. Once we begin recruiting from additional sites, we anticipate there may be more participants aged 16 to 18 years.

Eligibility is determined at the time of enrollment (T0). Participants need only identify as trans at the time of their surgery. Participants who no longer identify as trans at follow-up time points (T1 or T2) are still invited to complete follow-up questionnaires, as this represents valuable data regarding postsurgical experiences, outcomes, and trajectories.

#### Recruitment Process

**Figure 2.**
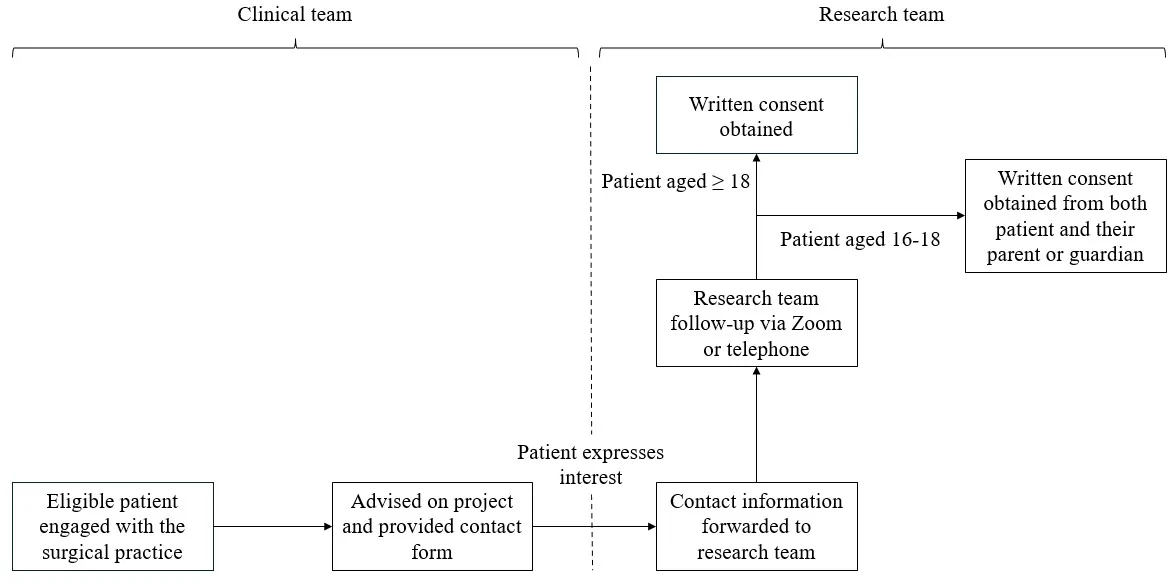
Schematic representation of the recruitment and consent process.

Prospective participants are approached by clinical staff and offered a contact form outlining a brief overview of the study, as well as space for the patient’s name and contact details if they are interested in participating or obtaining further information. Completed contact forms are then passed on to the research team by clinical staff; patients also have the option to email the research team directly. Interested individuals are sent information about the research and consent process via email, with the option to discuss the research further via videoconference or telephone. An overview of the recruitment and consent process is presented in Figure 2.

#### Data Collection

Participants are sent a link to complete each questionnaire at the beginning of each time point. Once the questionnaire has been sent, participants will have 2 weeks to complete the survey, after which the link will expire. Participants who have not completed the questionnaire, including those who have begun the questionnaire but not finished it, are sent a reminder via email 1 week after the survey was sent. The participating surgeon does not know which patients are participating until after their surgery, at which point the surgeon is asked to complete the patient’s surgical pro forma.

### Questionnaire Development and Testing

The questionnaires were developed through a multistage iterative process involving community consultation and professional input. Initial item development was informed by a review of existing literature on mental health, well-being, and surgical outcomes in trans populations and broader surgical contexts. A preliminary item pool was developed and refined by the research team, with the participating surgeon specifically reviewing the surgeon pro forma to ensure clinical relevance.

A Community Advisory Group, comprising 13 trans individuals who had recently undergone or were planning to undergo top surgery, convened twice during the development of the questionnaires. The group reviewed draft items and provided feedback that was used to refine item wording, ensure questions and response options were appropriate and inclusive, ensure all relevant topics were addressed, and identify areas not adequately covered by the included validated measures. This iterative process informed the development of bespoke items to address these gaps.

The final questionnaires combine validated measures, targeted bespoke items, and open-ended questions (refer to Multimedia Appendices 1-3). Validated instruments were selected based on their psychometric properties, relevance to the study’s outcomes of interest, and applicability to trans people, including prior use with trans populations where possible. Three members of the community advisory group tested the T0 questionnaire and their feedback was incorporated across all 3 questionnaires.

### Measures

#### Patient-Reported Outcome Measures

##### Overview

The following outcomes are measured within 8 weeks prior to surgery (T0), 6‐8 weeks post surgery (T1), and 1 year post surgery (T2), as self-reported by participants. Table 1 outlines which validated instruments will be used at each time point. To reduce participant burden during the postoperative period, some measures were excluded from the T1 questionnaire. The measures chosen for exclusion assess constructs that may change following surgery in general (ie, not specific to gender-affirming procedures), such as exercise, eating behaviors, and substance use.

**Table 1.** Overview of constructs measured using validated instruments at each time point.

Construct	Measure	Time point		
		T0	T1	T2
Depression	PHQ-9^a^	✓	✓	✓
Anxiety	GAD-7^b^	✓	✓	✓
Quality of life	AQoL-8D^c^	✓	✓	✓
Gender euphoria	GES^d^	✓	✓	✓
Chest dysphoria	Olson-Kennedy Scale^e^	✓	✓^f^	✓
Physical activity	IPAQ-SF^g^	✓		✓
Disordered eating	EDE-QS^h^	✓		✓
Substance use	ASSIST-Lite^i^	✓		✓
Surgeon satisfaction	GENDER-Q Surgeon Satisfaction subscale	✓	✓	✓

a PHQ-9: Patient Health Questionnaire-9.

b GAD-7: Generalized Anxiety Disorder 7-item scale.

c AQoL-8D: Assessment of Quality of Life - 8 Dimensions.

d GES: Gender Euphoria Scale.

e This scale has been modified for people undergoing breast augmentation surgery such that “chest” is replaced with “breast,” and an item on chest binding has been removed.

f Some items from this measure are excluded at this time point.

g IPAQ-SF: International Physical Activity Questionnaire - Short Form.

h EDE-QS: Eating Disorder Examination Questionnaire - Short.

i ASSIST-Lite: Alcohol, Smoking and Substance Involvement Screening Test - Lite.

##### Depression

Depression is measured with the Patient Health Questionnaire-9 (PHQ-9) [102]. The PHQ-9 is a validated 9-item scale based on *DSM-IV* (*Diagnostic and Statistical Manual of Mental Disorders* [Fourth Edition]) criteria that asks participants to report depressive symptoms over the past 2 weeks. Items are rated on a 4-point scale from 0 (not at all) to 3 (nearly every day). Scores of 0‐4 indicate no or minimal depression, 5‐9 indicate mild depression, 10‐14 indicate moderate depression, 15‐19 indicate moderately severe depression, and 20 or above indicate severe depression [102].

##### Anxiety

Anxiety is measured using the Generalized Anxiety Disorder 7-item scale (GAD-7) [103]. The GAD-7 uses a 4-point scale ranging from 0 (not at all) to 3 (nearly every day) to measure anxiety symptoms over the past 2 weeks. Scores of 0‐4 indicate minimal anxiety, 5‐9 indicate mild anxiety, 10‐14 indicate moderate anxiety, and 15 or above indicate severe anxiety [103].

##### Eating Behaviors and Disordered Eating

The Eating Disorder Examination Questionnaire - Short (EDE-QS), a 12-item version of the EDE-QS, is used to measure eating disorder symptoms and behaviors over the past week [104]. Items are rated from 0 to 3, with scores summed and averaged to calculate a total score and higher scores reflecting greater disordered eating [104]. Targeted items also assess the problematic use of eating and exercise behaviors to manage or ameliorate gender dysphoria specifically.

##### Substance Use

Substance use is measured using the Alcohol, Smoking, and Substance Involvement Screening Test - Lite (ASSIST-Lite) [105]. The ASSIST-Lite assesses the use of various substances over the past 3 months, with participants scoring from 0 to 3 for each substance and higher scores indicating greater risk of harm [105].

##### Gender Euphoria

Gender euphoria is measured using the GES [90]. The GES asks participants to rate scenarios over the past 2 weeks using a 5-point Likert scale from 1 (no gender euphoria) to 5 (very strong gender euphoria). The GES produces 4 scores: an overall scale score and subscale scores for social affirmation, self-affirmation, and community. Items are summed and averaged, with higher scores reflecting greater gender euphoria.

##### Chest Dysphoria

Chest dysphoria is measured using the Olson-Kennedy Scale [36]. This 11-item scale asks participants to rate statements based on their experiences over the past 2 weeks on a 4-point scale from 0 (never) to 4 (all the time). Scores range from 0 to 51, with higher scores indicating greater chest dysphoria [36]. The scale has been adapted for breast augmentation surgeries (eg, “chest” has been changed to “breast” and an item on chest binders has been removed). Additional targeted items have been added following the scale to capture nuanced experiences of chest dysphoria and gender dysphoria more broadly.

##### Quality of Life

Quality of life is measured using the Assessment of Quality of Life - 8 Dimensions (AQoL-8D) [106]. This multidimensional measure assesses experiences over the past week across 8 domains: independent living, pain, senses, mental health, happiness, coping, relationships, and self-worth. In addition, Australian normative data for each subscale enable comparisons with the general population [106].

##### Physical Activity

Physical activity is assessed using the International Physical Activity Questionnaire - Short Form (IPAQ-SF) [107]. This 7-item questionnaire records the time (in minutes) spent on vigorous-intensity activity, moderate-intensity activity, and walking over the past 7 days. Standard metabolic equivalent of task (MET) levels (walking: 3.3; moderate: 4.0; vigorous: 8.0) are used to calculate MET-minutes per week (MET-min/wk) for each activity type. These are calculated using the formula: MET-level × minutes per day × days per week. Scores can be reported either as continuous scores (total MET-min/wk) or categorically (high, moderate, or low) based on total scores and activity frequency [108].

##### Surgeon Satisfaction

Satisfaction with patient-surgeon interactions is measured using the 15-item Health Professional subscale of the GENDER-Q [93]. Participants rate their agreement with statements about recent surgeon interactions using a 4-point scale from 1 (strongly disagree) to 4 (strongly agree). Higher scores indicate greater satisfaction with patient-surgeon interactions.

##### Surgical Outcome Satisfaction

Surgical satisfaction is assessed via targeted questions addressing surgical complications (if applicable) and satisfaction with esthetic aspects such as chest contour, nipple areola complex esthetics, skin appearance, scarring, and overall appearance.

##### Additional Questions

In addition to the above validated instruments, bespoke questions were developed through community consultation and are used to assess plans and expectations for surgery, barriers to accessing surgery, support received throughout the surgery process, gender-affirming hormone use and other gender-affirming surgeries, experiences of self-harm and suicidality, current and past mental health diagnoses, and recent experiences of discrimination and social acceptance. Table 2 outlines which bespoke items are included at each time point. Some bespoke questions were also excluded at T1 either to reduce participant burden (eg, eating and exercise behaviors) or because they were unlikely to have changed from T0 (eg, experience with hormones and other surgeries, mental health, and neurodevelopmental diagnoses).

**Table 2.** Overview of constructs measured using bespoke questions at each time point.

Construct	Time point		
	T0	T1	T2
Planning and expectations for surgery	✓		
Barriers to accessing surgical care	✓		
Supports available and received throughout the surgery process	✓	✓	✓
Experiences with gender-affirming hormones and other surgeries	✓		✓
Satisfaction with surgical outcomes		✓	✓
Gender-related eating and exercise behaviors	✓		✓
Self-harm and suicidal thoughts and behaviors	✓	✓^a^	✓
Mental health and neurodevelopmental diagnoses	✓		✓
Social acceptance and discrimination	✓	✓	✓

a Some items assessing this construct were excluded from the questionnaire at this time point.

Open-ended questions are embedded throughout each questionnaire, providing participants the option to expand upon their responses to the quantitative questions, contextualize their experiences of accessing top surgery, and report on anything significant to them that wasn’t already captured by the existing measures. Responses are optional and may vary in length and depth. Examples of open-ended questions include “What strategies are you using, or have you been using, to cope while waiting for top surgery?” and “Do you feel your mental health has changed (positively or negatively) since having surgery? If so, please tell us how.”

##### Surgeon-Reported Outcome Measures

Surgeon pro formas are completed around the time of each patient’s follow-up appointment, estimated to occur approximately 3 months post surgery. Each pro forma includes the type of surgery performed, any postsurgical complications, an evaluation of scars (categorized as good, average, or poor), and any additional comments pertinent to the surgery. The pro forma was developed in consultation with the participating surgeon and is based on established surgical outcome standards.

Information from the participating surgeon will be used in exploratory analyses to examine the effects of these factors on outcomes. Surgeon-reported outcomes will also be compared to patient-reported outcomes to examine differences, as prior research suggests that patients rate their postsurgery chest appearance more favorably than their surgeons [109].

### Statistical Analyses

#### Quantitative Analysis

Descriptive statistics will be calculated for all continuous and categorical variables. Correlation analyses will be conducted to assess the associations between outcome variables and to identify any potential confounders that ought to be controlled in the main analyses (eg, age, gender, and assigned sex at birth). Difference scores between T0, T1, and T2 will be analyzed using 2-sided *t* tests and chi-square tests. Repeated-measures ANOVAs will be used to compare patient-reported and surgeon-reported satisfaction with surgical outcomes at T1 and T2. Linear regression analyses will be conducted to examine whether patient-reported satisfaction with surgical outcomes predicts mental health, substance use, disordered eating, gender euphoria, chest dysphoria, physical activity, and quality of life at T1 and T2. Regression models will be adjusted for relevant demographic variables. If additional surgeons are included in the study, “surgeon” will be added as a covariate. If there is an insufficient sample size for these analyses, we will investigate the use of alternative statistical methods, eg, Bayesian statistics. All quantitative analyses will be performed using SPSS (IBM Corp) [110] or RStudio (Posit PBC) [111], as appropriate.

#### Qualitative Analysis

Qualitative responses to open-ended questions will be analyzed in NVivo (Lumivero) [112] using the general inductive approach (GIA) [113]. The use of a low-theory, inductive analytic method such as GIA will enable us to derive categories directly from participants’ own words, without imposing a predefined theoretical framework. This approach allows us to capture participants’ experiences of accessing top surgery in a way that aligns closely with the constructs of interest, while remaining open to unexpected or novel insights that extend beyond our initial hypotheses. GIA allows for the development of clear, data-driven categories that contextualize and support the interpretation of quantitative findings and also permits the descriptive quantification of categories where useful for understanding the prevalence of particular experiences.

Each open-text question will be analyzed independently following the same process. After reading through the participant responses to become familiar with the content of the data, the analyzing researcher will identify and descriptively code each segment of meaning within the responses. Next, the analyzing researcher will begin to group codes together into preliminary categories before refining these categories to ensure each is conceptually coherent and distinct from the others. Once categories have been defined for each question, they will be mapped onto the theoretical construct they are relevant to, enabling further contextualization of the quantitative findings. If appropriate, categories will be quantified in order to illustrate the prevalence of certain experiences, particularly in the case of any novel or unexpected findings. Finally, categories within each construct will be compared across the 3 time points in order to understand how participants’ experiences change and develop over time.

In the interest of methodological rigor, the analyzing researcher will engage deeply with the data through a systematic and iterative approach to coding, keep a detailed audit trail of analytic decisions, and give due attention to any negative cases or divergent experiences. Supporting excerpts from the data will be reported to demonstrate credibility. The analyzing researcher will also engage in reflexive practices (eg, note-taking and journaling) and will discuss and debrief with other members of the research team throughout the analytic process.

### Risk Management

There is a chance that participation in any research may cause distress, particularly when reflecting on experiences related to gender-affirming care, mental health, or discrimination. This potential risk is outlined in the participant information sheet, which all participants are asked to read prior to consenting. A risk management protocol has been developed to manage risk if participants indicate thoughts of self-harm or suicide during the questionnaires or request further support from the research team. Prior to the mental health–related section of the questionnaire, a disclaimer advises participants that their responses will remain confidential (but not anonymous). Participants are also advised that if their responses indicate a high level of risk, a member of the research team will attempt to contact them to offer assistance in accessing mental health support. Mental health and crisis support service contact details are provided before and after the mental health sections and at the end of each questionnaire.

If a participant selects a response greater than 0 (not at all) on Item 9 (“Thoughts that you would be better off dead, or of hurting yourself”) of the PHQ-9, or anything greater than (never) on Item 16 (“Do you ever feel like hurting yourself?”) of the AQoL-8D, an automated email alert is sent to the research team. A member of the research team will then contact the participant via email to offer assistance in accessing additional support. If the participant requests follow-up, a member of the research team will contact them and administer the Columbia-Suicide Severity Rating Scale [114] and implement further support strategies depending on the level of risk identified. If the participant does not respond to the initial email, a member of the research team will follow up via text message. If the participant does not respond to the text message, a second email will be sent providing a comprehensive list of mental health and crisis support services. At this stage, it will be assumed that the participant does not want assistance from the research team.

There are minor risks associated with the surgeon knowing which patients have consented to participate in the study. Participants might feel pressured to participate based on their relationship with the surgeon or alter their responses due to concerns about how these will impact their medical care. To minimize this risk, all recruitment and consent procedures will be conducted independently of the surgical setting. Participants will be informed that their surgeon will not have access to their self-reported data at any stage, that their surgeon will only become aware of their study involvement after their surgery has been completed, and that choosing not to participate will not affect their medical care.

### Data Management

Patient-reported data will be collected online through the University of Western Australia’s secure Qualtrics server. Surgeon-reported data from pro formas will be manually entered into a deidentified digital database. Physical data will be stored in a locked cabinet at The Kids Research Institute Australia, and all digital data will be password-protected and stored on secure servers. As the study may include participants younger than the age of 18 years, data will be retained for a minimum of 7 years following the last publication or until all participants are aged 25 years, whichever occurs later. The findings of this study will be submitted for publication in a peer-reviewed journal and may also be presented at conferences nationally (eg, Australian Professional Association for Trans Health and Australian Society of Plastic Surgeons) and internationally (eg, World Professional Association for Trans Health).

## Results

This study obtained funding in February 2024. Data collection commenced in May 2025 and is expected to end in December 2027. As of February 2026, 48 patients had expressed interest in participating in the study, 26 had consented to participate, 15 had completed the T0 questionnaire, 6 had completed the T1 questionnaire, and none were yet eligible to complete the T2 questionnaire. Four surgeon pro formas had also been completed. Data analysis had not yet commenced. We anticipate that major results of this study will be published in 2029; preliminary results may be available sooner.

After commencing data collection, it became clear that our recruitment strategy was limiting how many participants we could recruit. Initially, participants needed to have a surgery date booked before consenting to participate in the study, and they would complete the T0 questionnaire 6‐8 weeks before their surgery. However, feedback from the surgeon’s office was that many participants did not have a surgery date booked immediately following their consultation, and that surgeries were often booked less than 6 weeks in advance, and so we were missing the opportunity to recruit many potential participants. We have since changed our recruitment strategy so that participants can complete the T0 questionnaire any time within 8 weeks prior to their surgery, and they do not need to have a surgery date booked in order to consent to participate. We also simplified the consent form so that instead of providing their contact details themselves, participants’ contact details are shared with the research team by the surgery staff.

## Discussion

### Strengths and Contributions

This prospective cohort study aims to extend existing research by comprehensively examining the long-term impacts of top surgery on mental health, dysphoria, and quality of life among trans people in Australia. Most existing research has been conducted in North America [49-52,54-56] and Europe [66,67,78], where health care systems differ substantially from the Australian context. Barriers to accessing top surgery vary internationally; in Australia, out-of-pocket cost is the primary barrier, yet the mental health implications of this specific access landscape remain underexplored. By collecting both quantitative and qualitative data, this study will provide Australia-specific evidence on postsurgical outcomes as well as important context regarding barriers to accessing care.

This study also addresses key methodological limitations of existing research by adopting a prospective longitudinal design, enabling direct comparison of patient-reported outcomes before and after surgery. Unlike the majority of previous research, which has used retrospective designs [51,64-67], this approach allows for more accurate assessment of changes over time and identification of specific temporal patterns. For instance, some participants could experience immediate improvements in their mental health, while others might face initial challenges, such as stress and postoperative pain, before experiencing longer-term mental health improvements. Understanding these patterns and exploring potential associations will help inform patient expectations, pre- and postoperative care, and support strategies.

An additional strength of this study is its use of validated instruments to measure patient-reported outcomes, several of which have been developed specifically for use in trans populations [36,90,93]. Using these measures should address limitations of past studies that relied on unvalidated or ad hoc measures [36,84,85,90,93], providing a more robust understanding of postsurgical outcomes. Moreover, the inclusion of nonbinary participants—a population often underrepresented in research on gender-affirming surgeries [64,74,94]—will provide novel insights into their specific preferences, experiences, and outcomes. This inclusion will also allow for direct comparison between binary and nonbinary participants, contributing to more inclusive and informed care for nonbinary people undergoing top surgery.

### Limitations

Several limitations concerning the proposed study should be acknowledged. First, as top surgery is inaccessible through the Australian public health system, access is currently limited to those with private health insurance or the financial means to self-fund their surgery. As a result, the study sample may not be representative of the broader trans population, specifically those who do not wish for top surgery or are unable to access it for financial or other reasons. It is likely that participants will have higher incomes or greater access to financial resources, potentially skewing results toward more favorable outcomes. For instance, a sample consisting of high-income earners might mean participants face fewer barriers, have greater access to material supports, or have better mental health overall [115-117].

Second, recruitment from a single surgical site may limit the generalizability of our findings. As the participating surgeon specializes in transverse inframammary incisions with free nipple grafting, or “double incision technique,” outcomes related to surgical satisfaction and healing may not be generalizable to other types of top surgery. Additionally, the study may overrepresent individuals seeking chest reconstructive procedures, similar to much of the existing research [118,119]. Planned expansion to additional surgical sites in the future is expected to address this limitation.

In addition, there is a risk of attrition bias in the completion of follow-up assessments. Participants with poorer mental health outcomes may be more likely to drop out between time points [120-122], which could lead to an overrepresentation of favorable experiences and an underestimation of potential challenges or negative outcomes. While studies suggest that selective attrition does not significantly bias results [123,124], we will nevertheless implement strategies to minimize disengagement. These include offering flexible completion options and sending multiple reminders to complete the follow-up questionnaires. In addition, we will continue to monitor attrition and consider implementing statistical methods, such as inverse probability weighting, if necessary.

Finally, the study follows participants for only 1 year post surgery. While this time frame is longer than that of many existing studies [68,69], it does not capture longer-term changes that may occur beyond the first year post surgery. Additionally, with only 2 follow-up periods, we may miss potential fluctuations in outcomes between these time points. The inclusion of consent to contact participants for additional follow-up questionnaires, however, allows for the possibility of longer-term follow-up in future phases of the project.

### Implications

The proposed study is expected to enrich and extend our understanding of top surgery outcomes for trans people, including nonbinary people, in Australia. Findings are anticipated to have substantial implications for the provision of gender-affirming health care, offering health care professionals—including surgeons, general practitioners, and mental health clinicians—a deeper understanding of patients’ needs and evidence to guide patient-centered care. The findings also have the potential to offer trans people considering surgery a clearer understanding of the potential impacts on mental health, dysphoria, and quality of life, thereby informing and empowering their decision-making.

By expanding upon previous research demonstrating the positive impacts of top surgery, this study has the potential to inform broader policy and public health initiatives related to gender-affirming care. Through contributing robust, longitudinal data, the findings of this study may support advocacy efforts to improve access to top surgery in Australia and promote the development of inclusive, affirming health care systems. For example, we will be able to conduct a cost-utility analysis using the quality-of-life data, which will be informative for the current process of consideration of top surgery for inclusion on the Medicare Benefits Schedule by determining the value of subsidizing the cost of this surgery.

### Conclusion

This study seeks to evaluate the impact of top surgery on trans individuals by comprehensively assessing mental health, dysphoria, and quality-of-life outcomes over time. It is expected that findings will guide improvement in surgical care and follow-up protocols, inform health care policy, and ultimately improve the quality and accessibility of care for trans people undergoing top surgery.

## Supplementary material

10.2196/83928 Multimedia Appendix 1 Questionnaire 8 weeks before surgery.

10.2196/83928 Multimedia Appendix 2 Questionnaire 6 weeks post surgery.

10.2196/83928 Multimedia Appendix 3 Questionnaire 1 year post surgery.

## Abbreviations

AQoL-8D: Assessment of Quality of Life - 8 Dimensions
ASSIST-Lite: Alcohol, Smoking and Substance Involvement Screening Test - Lite
*DSM-5-TR*: *Diagnostic and Statistical Manual of Mental Disorders, Fifth Edition, Text Revision*
*DSM-IV*: *Diagnostic and Statistical Manual of Mental Disorders [Fourth Edition]*
EDE-QS: Eating Disorder Examination Questionnaire - Short
GAD-7: Generalized Anxiety Disorder Assessment - 7 item scale
GES: Gender Euphoria Scale
GIA: general inductive approach
*ICD-11*: *International Statistical Classification of Diseases and Related Health Problems, Eleventh Revision*
IPAQ-SF: International Physical Activity Questionnaire - Short Form
MET: metabolic equivalent of task
PHQ-9: Patient Health Questionnaire-9
