# Supplementary material for: Mental Health, Gender Dysphoria, and Quality of Life in Trans Individuals Following Top Surgery: Protocol for a Prospective Cohort Study
Source: JMIR Res Protoc. 2026 Aug 21;15:e83928. doi: 10.2196/83928 (PMC13496940; doi:10.2196/83928)
Supplement: Supplementary file 2 [file resprot-v15-e83928-s002.docx]

**TOP SURGERY STUDY**

**6 WEEKS POST-SURGERY QUESTIONNAIRE**

Welcome to the Top Surgery Study 6 weeks post-surgery questionnaire. Thank you for consenting to take part in this study.

This study aims to assess the impacts of top surgery (an umbrella term that includes both chest reconstructive surgery and breast augmentation) on the gender dysphoria, mental health, well-being, and quality of life of trans and gender diverse individuals over time. For more information about the research project and the information we want to collect, please refer to the Participant Information Sheet.

If you do not feel able to complete parts of this questionnaire at this time, please feel free to skip any questions you do not feel comfortable answering. You can also choose to withdraw from the study at any point.

We recognise this is a long survey, so please take your time in completing it and come back to it as needed. This questionnaire link is unique to your email address. This means that you can take breaks from the questionnaire rather than having to complete it all at once, provided you access the link using the same device.

This questionnaire link will be active for two weeks from the date we emailed it to you. We will send you a reminder to complete the survey after one week. If you have any questions, please contact us via email at [topsurgery@thekids.org.au](mailto:topsurgery@thekids.org.au).

# DEMOGRAPHICS This first set of questions are about you, so that we can accurately describe everyone involved in this study.

| **Questions** | **Variables** | **6 wk pre** | **6 wk post** | **1 yr post** |
| --- | --- | --- | --- | --- |
| 1. **[mandatory response]** What is your last name?   *We are asking this to link your survey responses to your medical records. Your surgeon will not see your individual responses to this questionnaire.* *If your last name has changed since your last questionnaire, please let us know your current and previous last names.* | [open text] |  |  |  |
| 2. **[mandatory response]** What is your date of birth?   *We will ask you this on each survey to link your responses.* | - Day/month/year |  |  |  |
| 3. What is your current preferred contact email? | [open text] |  |  |  |
| 4. **[mandatory response]** Would you prefer us to use “chest” or “breast/s” throughout this survey?  *For example, the following question will be phrased as either: “If you experience chest dysphoria, has your chest dysphoria changed since booking surgery?” or “If you experience breast dysphoria, has your breast dysphoria changed since booking surgery?* | - Chest - Breast/s |  |  |  |
| 5. How do you describe your gender (e.g. woman, man, trans man, brotherboy, bigender, ambigender, demigirl)?   *Feel free to put in more than one identifying term if relevant to you.* | [open text] |  |  |  |
| 6. For the purposes of data analysis we sometimes have to use gender categories. Which of the following terms best describes you: | - Man or male - Woman or female - Non-binary |  |  |  |
| 7. How do you describe your sexuality? Feel free to put in more than one identifying term if relevant to you. | [open text] |  |  |  |
| 8. What best describes your current living situation? (Select all that apply) | - On my own - With a spouse/partner/s - With my parent/s - With other family members - Foster home - Share house - Residential college - Boarding house - Supported accommodation (e.g., Foyer Oxford) - Assisted living/care facility - No fixed accommodation - Prefer not to say - Other, please specify [open text] |  |  |  |
| 9. What is the postcode where you currently live/spend most of your time? | [open text] |  |  |  |
| 10. What type of surgery did you have? | - Double incision, nipple graft - Double incision, no nipples - Periareolar (a cut made around the whole nipple); nipple resized - Periareolar (a cut made around the whole nipple); nipple not resized - Keyhole/hemiareolar (a cut made under the nipple); nipple resized - Keyhole/hemiareolar (a cut made under the nipple); nipple not resized - Breast reduction (often from a larger cup size to a smaller cup size, with some tissue remaining) - Breast augmentation, implant placed over the pectoral muscle - Breast augmentation, implant placed under the pectoral muscle - Prefer not to say - Other, please specify: [open text] |  |  |  |
| 11. At the time of surgery, did you have private health insurance? | - Yes - No - Prefer not to say |  |  |  |
| 12. At the time of surgery, what gender marker was recorded with Medicare?  *We are asking this so that we can calculate the cost of your surgery.* | - Male - Female - Prefer not to say |  |  |  |

# GENERAL SUPPORT The next few questions ask about the support you received when making decisions about and accessing your top surgery.

# In the time since having top surgery, how often have you felt supported by your:

| **Questions** | **Variables** | **6 wk pre** | **6 wk post** | **1 yr post** |
| --- | --- | --- | --- | --- |
| 13. Partner/s | - Never - Sometimes - Frequently - All the time - Does not apply to me - Prefer not to say |  |  |  |
| 14. Family | - Never - Sometimes - Frequently - All the time - Does not apply to me - Prefer not to say |  |  |  |
| 15. Friends | - Never - Sometimes - Frequently - All the time - Does not apply to me - Prefer not to say |  |  |  |
| 16. Surgeon | - Never - Sometimes - Frequently - All the time - Does not apply to me - Prefer not to say |  |  |  |
| 17. Work | - Never - Sometimes - Frequently - All the time - Does not apply to me - Prefer not to say |  |  |  |
| 18. University | - Never - Sometimes - Frequently - All the time - Does not apply to me - Prefer not to say |  |  |  |
| 19. Who or what have been the most important sources of support for your mental health following your top surgery? (Select all that apply) | - Immediate family (e.g., siblings, parents) - Extended family (e.g., cousins, grandparents) - Friends/peers - Other trans people - My partner/s - Mental health professional/s - GP - Online groups (e.g., Discord) - Peer support groups (e.g., TransFolk of WA) - None of the above - Prefer not to say - Other, please specify: [open text] |  |  |  |
| 20. From your surgery day onwards, do you feel you have had enough support from the people in your life? | - Yes - No - Prefer not to say |  |  |  |
| 21. Was it difficult to find a support person to help you during the first 2 weeks of recovery? | - Yes - No - Prefer not to say |  |  |  |
| 22. Since having surgery, if you have accessed mental health support, what types have you accessed? (Select all that apply) | - Peer support (either online or face to face) - Therapy (either online or face to face) - GP or another medical specialist - I have not accessed mental health support - Prefer not to say - Other, please specify: [open text] |  |  |  |
| 23. How could mental health support before and post-surgery be improved? (Select all that apply) | - GP’s need to be informed about mental health needs of trans people going through this experience - Therapists need to be more informed about mental health needs of trans people going through this experience - More face-to face local peer support - More online local peer support - The mental health support I received was adequate - Prefer not to say - Other, please specify: [open text] |  |  |  |
| 24. Did you feel appropriately informed about the healing and recovery process prior to surgery? | - Yes - No - Unsure - Prefer not to say |  |  |  |
| 25. After top surgery, you’re usually given a variety of pain killers to take home with you. This may pose challenges for people struggling with addiction and substance abuse, or who live with a person struggling with addiction and substance abuse. Was this a concern for you? | - Yes, it was a major concern - Yes, it was a minor concern - No, it wasn’t a concern - Prefer not to say |  |  |  |
| 26. *If yes,* did you have adequate resources to manage/respond to this situation?   *Will only display if ‘yes, it was a major concern’* ***or*** *‘yes, it was a minor concern’ is selected in response to the previous question.* | - Yes - No - Prefer not to say - Other, please specify: [open text] |  |  |  |
| 27. Post-surgery, how long were you experiencing pain that interfered with daily tasks? | - 1-3 days - 4-6 days - 1 week - 2 weeks - 3 weeks - 4 weeks - 5 weeks - 6 weeks + - Prefer not to say |  |  |  |
| 28. What was your experience of wearing the post op binder or surgical bra? (Select all that apply) | - It felt protective - It didn’t bother me - It was comfortable - I found it difficult to sleep in - I found it difficult to breathe - I felt trapped in it - It made me feel anxious - I’m sensitive to materials and wearing it was distressing - I overheated regularly - It was painful - It gave me a headache - It pulled down on my shoulders - It caused skin irritation/rash/rubbing - Prefer not to say - Other, please specify: [open text] |  |  |  |
| 29. Since having top surgery, what are some of the positive and exciting feelings you have had about your chest/breasts? | [open text] |  |  |  |

**GENDER-Q – HEALTH PROFESSIONAL**

These questions ask about the gender-affirming care you received from your surgeon. Please answer thinking of your most recent interactions. How much do you disagree or agree

that your surgeon:

| **Questions** | **Variables** | **6 wk pre** | **6 wk post** | **1 yr post** |
| --- | --- | --- | --- | --- |
| 30. Treated you with respect? | - Dissatisfied - Somewhat satisfied - Very satisfied - Extremely satisfied |  |  |  |
| 31. Made you feel safe? | - Dissatisfied - Somewhat satisfied - Very satisfied - Extremely satisfied |  |  |  |
| 32. Was trustworthy? | - Dissatisfied - Somewhat satisfied - Very satisfied - Extremely satisfied |  |  |  |
| 33. Made you feel affirmed in your gender identity? | - Dissatisfied - Somewhat satisfied - Very satisfied - Extremely satisfied |  |  |  |
| 34. Asked you questions that were relevant to the treatment you were seeking? | - Dissatisfied - Somewhat satisfied - Very satisfied - Extremely satisfied |  |  |  |
| 35. Helped you access the treatment you need? | - Dissatisfied - Somewhat satisfied - Very satisfied - Extremely satisfied |  |  |  |
| 36. Answered all your questions? | - Dissatisfied - Somewhat satisfied - Very satisfied - Extremely satisfied |  |  |  |
| 37. Involved you in decisions about your treatments? | - Dissatisfied - Somewhat satisfied - Very satisfied - Extremely satisfied |  |  |  |
| 38. Listened to you and understood your concerns? | - Dissatisfied - Somewhat satisfied - Very satisfied - Extremely satisfied |  |  |  |
| 39. Was easy to talk to? | - Dissatisfied - Somewhat satisfied - Very satisfied - Extremely satisfied |  |  |  |
| 40. Helped you have realistic expectations about gender-affirming treatments? | - Dissatisfied - Somewhat satisfied - Very satisfied - Extremely satisfied |  |  |  |
| 41. Provided the highest possible level of care? | - Dissatisfied - Somewhat satisfied - Very satisfied - Extremely satisfied |  |  |  |
| 42. Was thorough? | - Dissatisfied - Somewhat satisfied - Very satisfied - Extremely satisfied |  |  |  |
| 43. Explained treatment options in as much detail as you would like? | - Dissatisfied - Somewhat satisfied - Very satisfied - Extremely satisfied |  |  |  |
| 44. Helped you figure out what was best for you? | - Dissatisfied - Somewhat satisfied - Very satisfied - Extremely satisfied |  |  |  |
| 45. Please tell us more about this if you would like to: | [open text] |  |  |  |

**MENTAL HEALTH**

We would like to ask you about your mental health and wellbeing. This includes questions about suicide and self-harm. We understand that these experiences can be common. This research and your responses are confidential. If you are in a high-risk situation, we will attempt to contact you to assist you in getting support. We will not share your responses with anyone else or contact anyone on your behalf without your consent.

*If any of the following questions upset you, we urge you to contact Qlife LGBTI phone and chat counselling and info service (1800 184 527 www.qlife.org.au), Lifeline (13 11 14 www.lifeline.org.au), Suicide Call Back Service (1300 659 467* [*www.suicidecallbackservice.org.au/*](http://www.suicidecallbackservice.org.au/)*), beyondblue (1300 22 4636* [*www.beyondblue.org.au*](http://www.beyondblue.org.au)*), Kids Helpline (1800 55 1800* [*www.kidshelpline.com.au*](http://www.kidshelpline.com.au)*), 13YARN (13 92 76* [*www.13yarn.com.au*](http://www.13yarn.com.au)*) or any other support service.*

**ANXIETY – GAD-7**

Over the past 2 weeks, how often have you been bothered by any of the following problems?

| **Questions** | **Variables** | **6 wk pre** | **6 wk post** | **1 yr post** |
| --- | --- | --- | --- | --- |
| 46. Feeling nervous, anxious or on edge | - Not at all - Several days - More than half the days - Nearly every day |  |  |  |
| 47. Not being able to stop or control worrying | - Not at all - Several days - More than half the days - Nearly every day |  |  |  |
| 48. Worrying too much about different things | - Not at all - Several days - More than half the days - Nearly every day |  |  |  |
| 49. Trouble relaxing | - Not at all - Several days - More than half the days - Nearly every day |  |  |  |
| 50. Being so restless that it is hard to sit still | - Not at all - Several days - More than half the days - Nearly every day |  |  |  |
| 51. Becoming easily annoyed or irritable | - Not at all - Several days - More than half the days - Nearly every day |  |  |  |
| 52. Feeling afraid as if something awful might happen | - Not at all - Several days - More than half the days - Nearly every day |  |  |  |
| 53. If you checked off any problems, how difficult have these problems made it for you to do your work, take care of things at home, or get along with other people? | - Not difficult at all - Somewhat difficult - Very difficult - Extremely difficult |  |  |  |

**DEPRESSION – PHQ-9**

Over the past 2 weeks, how often have you been bothered by any of the following problems?

| **Questions** | **Variables** | **6 wk pre** | **6 wk post** | **1 yr post** |
| --- | --- | --- | --- | --- |
| 54. Little interest or pleasure in doing things | - Not at all - Several days - More than half the days - Nearly every day |  |  |  |
| 55. Feeling down, depressed, or hopeless | - Not at all - Several days - More than half the days - Nearly every day |  |  |  |
| 56. Trouble falling or staying asleep, or sleeping too much | - Not at all - Several days - More than half the days - Nearly every day |  |  |  |
| 57. Feeling tired or having little energy | - Not at all - Several days - More than half the days - Nearly every day |  |  |  |
| 58. Poor appetite or overeating | - Not at all - Several days - More than half the days - Nearly every day |  |  |  |
| 59. Feeling bad about yourself or that you are a failure or have let yourself or your family down | - Not at all - Several days - More than half the days - Nearly every day |  |  |  |
| 60. Trouble concentrating on things, such as reading the newspaper or watching television | - Not at all - Several days - More than half the days - Nearly every day |  |  |  |
| 61. Moving or speaking so slowly that other people could have noticed / Or the opposite, being so fidgety or restless that you have been moving around a lot more than usual | - Not at all - Several days - More than half the days - Nearly every day |  |  |  |
| 62. Thoughts that you would be better off dead, or of hurting yourself | - Not at all - Several days - More than half the days - Nearly every day |  |  |  |
| 63. If you checked off any problems, how difficult have these problems made it for your to do your work, take care of things at home, or get along with other people? | - Not difficult at all - Somewhat difficult - Very difficult - Extremely difficult |  |  |  |

**SELF-HARM AND SUICIDE**

| **Questions** | **Variables** | **6 wk pre** | **6 wk post** | **1 yr post** |
| --- | --- | --- | --- | --- |
| 64. Since your surgery have you wanted to harm yourself (without wanting to end your life)? | - Yes - No - Prefer not to say |  |  |  |
| 65. Since your surgery have you harmed yourself (without wanting to end your life)? | - Yes - No - Prefer not to say |  |  |  |
| 66. Since your surgery have you had serious thoughts about ending your life? | - Yes - No - Prefer not to say |  |  |  |
| 67. Since your surgery have you tried to kill yourself or made a suicide attempt? | - Yes - No - Prefer not to say |  |  |  |
| 68. Since your surgery have you deliberately done something reckless to purposely risk your life? | - Yes - No - Prefer not to say |  |  |  |

*If any of the previous questions caused you distress, please contact Qlife LGBTI phone and chat counselling and info service (1800 184 527 www.qlife.org.au), Lifeline (13 11 14 www.lifeline.org.au), Suicide Call Back Service (1300 659 467* [*www.suicidecallbackservice.org.au/*](http://www.suicidecallbackservice.org.au/)*), beyondblue (1300 22 4636* [*www.beyondblue.org.au*](http://www.beyondblue.org.au)*), Kids Helpline (1800 55 1800* [*www.kidshelpline.com.au*](http://www.kidshelpline.com.au)*), 13YARN (13 92 76* [*www.13yarn.com.au*](http://www.13yarn.com.au)*) or any other support service.*

**GENDER EUPHORIA SCALE (GES)**

Gender euphoria is a positive feeling about your gender identity, gender expression, or gender affirmation. Gender euphoria may involve feeling happy, confident, content, safe, or another feeling you recognise as being positive to you. Gender euphoria may arise when you feel good about your gender, when your gender is accepted and affirmed by yourself or others, or when you feel true to yourself and your gender.

The next series of questions ask about your experiences of gender euphoria. On a scale from 1 – 5, where 1 = no gender euphoria and 5 = very strong gender euphoria, how strong has your gender euphoria felt in the following scenarios over the past two weeks:

| **Questions** | **Variables** | **6 wk pre** | **6 wk post** | **1 yr post** |
| --- | --- | --- | --- | --- |
| 69. When people use my correct pronouns | - No gender euphoria - Slight gender euphoria - Moderate gender euphoria - Strong gender euphoria - Very strong gender euphoria - This scenario does not apply to me (NA) |  |  |  |
| 70. When I look in the mirror | - No gender euphoria - Slight gender euphoria - Moderate gender euphoria - Strong gender euphoria - Very strong gender euphoria - This scenario does not apply to me (NA) |  |  |  |
| 71. When my gender is affirmed in   everyday encounters (e.g., at shops, restaurants) | - No gender euphoria - Slight gender euphoria - Moderate gender euphoria - Strong gender euphoria - Very strong gender euphoria - This scenario does not apply to me (NA) |  |  |  |
| 72. When I see myself represented   in the workplace | - No gender euphoria - Slight gender euphoria - Moderate gender euphoria - Strong gender euphoria - Very strong gender euphoria - This scenario does not apply to me (NA) |  |  |  |
| 73. When people treat me the way   I want to be treated, in relation to my gender | - No gender euphoria - Slight gender euphoria - Moderate gender euphoria - Strong gender euphoria - Very strong gender euphoria - This scenario does not apply to me (NA) |  |  |  |
| 74. When my gender is affirmed at   work | - No gender euphoria - Slight gender euphoria - Moderate gender euphoria - Strong gender euphoria - Very strong gender euphoria - This scenario does not apply to me (NA) |  |  |  |
| 75. When I see my face in the mirror | - No gender euphoria - Slight gender euphoria - Moderate gender euphoria - Strong gender euphoria - Very strong gender euphoria - This scenario does not apply to me (NA) |  |  |  |
| 76. When I see transgender, gender   diverse, or non-binary people represented in the media | - No gender euphoria - Slight gender euphoria - Moderate gender euphoria - Strong gender euphoria - Very strong gender euphoria - This scenario does not apply to me (NA) |  |  |  |
| 77. When people use the correct   language to describe my gender | - No gender euphoria - Slight gender euphoria - Moderate gender euphoria - Strong gender euphoria - Very strong gender euphoria - This scenario does not apply to me (NA) |  |  |  |
| 78. When I connect with other   transgender, gender diverse, or non-binary people online | - No gender euphoria - Slight gender euphoria - Moderate gender euphoria - Strong gender euphoria - Very strong gender euphoria - This scenario does not apply to me (NA) |  |  |  |
| 79. When I see people who   experience their gender in a similar way to me | - No gender euphoria - Slight gender euphoria - Moderate gender euphoria - Strong gender euphoria - Very strong gender euphoria - This scenario does not apply to me (NA) |  |  |  |
| 80. When strangers affirm my gender in public | - No gender euphoria - Slight gender euphoria - Moderate gender euphoria - Strong gender euphoria - Very strong gender euphoria - This scenario does not apply to me (NA) |  |  |  |
| 81. When I see my facial hair (or lack thereof) | - No gender euphoria - Slight gender euphoria - Moderate gender euphoria - Strong gender euphoria - Very strong gender euphoria - This scenario does not apply to me (NA) |  |  |  |
| 82. When my gender is affirmed in   education settings | - No gender euphoria - Slight gender euphoria - Moderate gender euphoria - Strong gender euphoria - Very strong gender euphoria - This scenario does not apply to me (NA) |  |  |  |
| 83. When my gender is legally   recognised | - No gender euphoria - Slight gender euphoria - Moderate gender euphoria - Strong gender euphoria - Very strong gender euphoria - This scenario does not apply to me (NA) |  |  |  |
| 84. When I feel connected to the   transgender, gender diverse, and non-binary community | - No gender euphoria - Slight gender euphoria - Moderate gender euphoria - Strong gender euphoria - Very strong gender euphoria - This scenario does not apply to me (NA) |  |  |  |
| 85. When I access spaces that   affirm my gender (e.g., my workplace, parties) | - No gender euphoria - Slight gender euphoria - Moderate gender euphoria - Strong gender euphoria - Very strong gender euphoria - This scenario does not apply to me (NA) |  |  |  |
| 86. When I see myself represented   in the community | - No gender euphoria - Slight gender euphoria - Moderate gender euphoria - Strong gender euphoria - Very strong gender euphoria - This scenario does not apply to me (NA) |  |  |  |
| 87. When I see my body in the   mirror | - No gender euphoria - Slight gender euphoria - Moderate gender euphoria - Strong gender euphoria - Very strong gender euphoria - This scenario does not apply to me (NA) |  |  |  |
| 88. When I am welcomed into   spaces designated for my gender | - No gender euphoria - Slight gender euphoria - Moderate gender euphoria - Strong gender euphoria - Very strong gender euphoria - This scenario does not apply to me (NA) |  |  |  |
| 89. When my gender is affirmed by my family of origin (e.g., parents, guardians, siblings) | - No gender euphoria - Slight gender euphoria - Moderate gender euphoria - Strong gender euphoria - Very strong gender euphoria - This scenario does not apply to me (NA) |  |  |  |
| 90. When I am included by others   who have the same gender as me | - No gender euphoria - Slight gender euphoria - Moderate gender euphoria - Strong gender euphoria - Very strong gender euphoria - This scenario does not apply to me (NA) |  |  |  |
| 91. When my gender is affirmed by   strangers | - No gender euphoria - Slight gender euphoria - Moderate gender euphoria - Strong gender euphoria - Very strong gender euphoria - This scenario does not apply to me (NA) |  |  |  |
| 92. When I discuss my gender in   online forums (e.g., Reddit, Facebook groups) | - No gender euphoria - Slight gender euphoria - Moderate gender euphoria - Strong gender euphoria - Very strong gender euphoria - This scenario does not apply to me (NA) |  |  |  |
| 93. When I connect with other   transgender, gender diverse, or non-binary people in person | - No gender euphoria - Slight gender euphoria - Moderate gender euphoria - Strong gender euphoria - Very strong gender euphoria - This scenario does not apply to me (NA) |  |  |  |
| 94. When my identity documents (e.g., passport, driver’s license) accurately reflect my gender | - No gender euphoria - Slight gender euphoria - Moderate gender euphoria - Strong gender euphoria - Very strong gender euphoria - This scenario does not apply to me (NA) |  |  |  |

# CHEST/BREAST DYSPHORIA – OLSON-KENNEDY SCALE

Some trans and gender diverse people experience dysphoria. Dysphoria can be described as the distress experienced when your sex presumed at birth and gender identity do not align. Dysphoria may be associated with certain parts of the body, cultural expectations of gender and/or how others perceive you.

The following questions are about your feelings in the last two weeks.

| **Questions** | **Variables** | **6 wk pre** | **6 wk post** | **1 yr post** |
| --- | --- | --- | --- | --- |
| 95. I like looking at my chest/breasts in the mirror   *(Original question: I like looking at my chest in the mirror. The option “Prefer not to say” has been added.)* | - Never - Sometimes - Frequently - All the time - Prefer not to say |  |  |  |
| 96. I get misgendered because of my chest/breasts   *(Original question: I get gendered as female because of my chest. The option “Prefer not to say” has been added.)* | - Never - Sometimes - Frequently - All the time - Prefer not to say |  |  |  |
| 97. I have struggled to make future plans because of my chest/breasts   *(Original question: I have struggled to make future plans because of my chest. The option “Prefer not to say” has been added.)* | - Never - Sometimes - Frequently - All the time - Prefer not to say |  |  |  |
| 98. I feel like my life hasn’t started because of my chest/breasts   *(Original question: I feel like my life hasn’t started because of my chest. The option “Prefer not to say” has been added.)* | - Never - Sometimes - Frequently - All the time - Prefer not to say |  |  |  |
| 99. I worry that people are looking at my chest/breasts   *(Original question: I worry that people are looking at my chest. The option “Prefer not to say” has been added.)* | - Never - Sometimes - Frequently - All the time - Prefer not to say |  |  |  |
| 100. I participate in life less than others because of my chest/breasts   *(Original question: I participate in life less than others because of my chest. The option “Prefer not to say” has been added.)* | - Never - Sometimes - Frequently - All the time - Prefer not to say |  |  |  |
| 101. I feel connected to/at ease with my chest/breasts   (*The option “Prefer not to say” has been added.)* | - Never - Sometimes - Frequently - All the time - Prefer not to say |  |  |  |
| 102. In the last 3 weeks, I have enjoyed expressing my gender through clothing   (*The option “Prefer not to say” has been added.)* | - Never - Sometimes - Frequently - All the time - Prefer not to say |  |  |  |
| 103. I like looking at my chest/breasts with clothing on   (*The option “Prefer not to say” has been added.)* | - Never - Sometimes - Frequently - All the time - Prefer not to say |  |  |  |
| 104. I feel confident in my body   (*The option “Prefer not to say” has been added.)* | - Never - Sometimes - Frequently - All the time - Prefer not to say |  |  |  |

**DYSPHORIA – ADDITIONAL QUESTIONS**

| **Questions** | **Variables** | **6 wk pre** | **6 wk post** | **1 yr post** |
| --- | --- | --- | --- | --- |
| 105. How often have you experienced chest/breast dysphoria within the last two weeks? | - Never - Rarely - Sometimes - Often - Always - Prefer not to say |  |  |  |
| 106. *If you experience chest/breast dysphoria*, has your chest/breast dysphoria changed since having surgery?   *Will only display if something other than ‘never’ or ‘prefer not to say’ is selected in response to the previous question.* | - My chest/breast dysphoria has increased since having surgery - My chest/breast dysphoria has decreased since having surgery - My chest/breast dysphoria has stayed the same since having surgery - Does not apply to me - Prefer not to say - Other, please specify: [open text] |  |  |  |
| 107. *If you are currently on gender-affirming hormones,* what has impacted your chest/breast dysphoria more? | - Hormones - Surgery - Hormones and surgery have had an equal impact - Unsure - Does not apply to me - Prefer not to say |  |  |  |
| 108. Is there anything else you would like to tell us about your experience of chest/breast dysphoria? | [open text] |  |  |  |
| 109. Do you currently experience dysphoria in other parts of your body (other than chest/breasts)?  *By currently we are asking about the last two weeks.* | - Never - Rarely - Sometimes - Often - Always - Prefer not to say |  |  |  |
| 110. *If you currently experience dysphoria,* has your dysphoria increased in other parts of your body since surgery?   *Will only display if something other than ‘never’ or ‘prefer not to say’ is selected in response to the previous question.* | - I’m experiencing more dysphoria in other parts of my body - I’m experiencing dysphoria in parts of my body I wasn’t before - Does not apply to me - Prefer not to say |  |  |  |
| 111. If there is anything else you would like to tell us about your experience of dysphoria please do so here: | [open text] |  |  |  |
| 112. What strategies have you used to stay positive during top surgery recovery? | [open text] |  |  |  |
| 113. Can you tell us what it is about these things that make you feel better? | [open text] |  |  |  |

QUALITY OF LIFE – AQoL-8D

The following questions are about your general health and quality of life. These questions are from a measure used to look at quality of life. This information gives us insight into how top surgery impacts trans people’s day-to-day quality of life over time.

Select the option that best describes your situation over the last week.

| **Questions** | **Variables** | **6 wk pre** | **6 wk post** | **1 yr**  **post** |
| --- | --- | --- | --- | --- |
| 114. How much energy do you have to do the things you want to do? I am: | - Always full of energy - Usually full of energy - Occasionally energetic - Usually tired and lacking energy - Always tired and lacking energy |  |  |  |
| 115. How often do you feel socially excluded or left out? | - Never - Rarely - Sometimes - Often - Always |  |  |  |
| 116. How easy or difficult is it for you to get around by yourself outside your place of residence (e.g., shopping, visiting)? | - Getting around is enjoyable and easy - I have no difficulty getting around outside my house - A little difficulty - Moderate difficulty - A lot of difficulty - I cannot get around unless somebody is there to help me |  |  |  |
| 117. Does your health affect your role in your community (e.g., residential, sporting, church, or cultural activities)? | - My role in the community is unaffected by my health - There are some parts of my community role I cannot carry out - There are many parts of my community role I cannot carry out - I cannot carry out any part of my community role |  |  |  |
| 118. How often do you feel sad? | - Never - Rarely - Some of the time - Usually - Nearly all the time |  |  |  |
| 119. How often do you experience serious pain? I experience it: | - Very rarely - Less than once a week - Three to four times a week - Most of the time |  |  |  |
| 120. How much confidence do you have in yourself? | - Complete confidence - A lot - A moderate amount - A little - None at all |  |  |  |
| 121. Do you normally feel calm and tranquil or agitated? I am: | - Always calm and tranquil - Usually calm and tranquil - Sometimes calm and tranquil, sometimes agitated - Usually agitated - Always agitated |  |  |  |
| 122. Does your health affect your relationship with your family? | - My role in the family is unaffected by my health - There are some parts of my family role I cannot carry out - There are many parts of my family role I cannot carry out - I cannot carry out any part of my family role |  |  |  |
| 123. How satisfying are your close relationships (family and friends)? | - Very satisfying - Satisfying - Neither satisfying nor dissatisfying - Dissatisfying - Unpleasant - Very unpleasant |  |  |  |
| 124. How well do you communicate with others (talking, signing, texting, being understood by others and understanding them)? | - I have no trouble speaking to them or understanding what they are saying - I have some difficulty being understood by people who do not know me / I have no trouble understanding what others are saying to me - I am understood only by people who know me well / I have great trouble understanding what others are saying to me - I cannot adequately communicate with others |  |  |  |
| 125. How often do you have trouble sleeping? | - Never - Almost never - Sometimes - Often - All the time |  |  |  |
| 126. How often do you feel worthless? | - Never - Almost never - Sometimes - Usually - Always |  |  |  |
| 127. How often do you feel angry? | - Never - Almost never - Sometimes - Often - All the time |  |  |  |
| 128. How easy or difficult is it for you to move around (using any aids or equipment you need e.g., a wheelchair, frame, or stick)? | - I am very mobile - I have no difficulty with mobility - I have some difficulty with mobility (for example, going uphill) - I have difficulty with mobility / I can go short distance only - I have a lot of difficulty with mobility / I need someone to help me - I am bedridden |  |  |  |
| 129. Do you ever feel like hurting yourself? | - Never - Almost never - Sometimes - Often - All the time |  |  |  |
| 130. How enthusiastic do you feel? | - Extremely - Very - Somewhat - Not much - Not at all |  |  |  |
| 131. How often did you feel worried in the last seven days? | - Never - Occasionally - Sometimes - Often - All the time |  |  |  |
| 132. How difficult is it for you to wash, toilet, dress yourself, eat or care for your appearance? | - These tasks are very easy for me - I have no real difficulty in carrying out these tasks - I find some of these tasks difficult, but I manage to do them on my own - Many of these tasks are difficult, and I need help to do them - I cannot do these tasks by myself at all |  |  |  |
| 133. How often do you feel happy? | - All the time - Mostly - Sometimes - Almost never - Never |  |  |  |
| 134. How much do you feel you can cope with life’s problems? | - Completely - Mostly - Partly - Very little - Not at all |  |  |  |
| 135. How much pain or discomfort do you experience? | - None at all - I have moderate pain - I suffer from severe pain - I suffer unbearable pain |  |  |  |
| 136. How much do you enjoy your close relationships (family and friends)? | - Immensely - A lot - A little - Not much - I hate it |  |  |  |
| 137. How often does pain interfere with your usual activities? | - Never - Rarely - Sometimes - Often - Always |  |  |  |
| 138. How often do you feel pleasure? | - Always - Usually - Sometimes - Almost never - Never |  |  |  |
| 139. How much of a burden do you feel you are to other people? | - Not at all - A little - A moderate amount - A lot - Totally |  |  |  |
| 140. How content are you with your life? | - Extremely - Mainly - Moderately - Slightly - Not at all |  |  |  |
| 141. How well can you see (using your glasses or contact lenses if they are needed)? | - I have excellent sight - I see normally - I have some difficulty focusing on things, or I do not see them sharply e.g., small print, a newspaper or seeing objects in the distance - I have a lot of difficulty seeing things / My vision is blurred / I can see just enough to get by with - I only see general shapes / I need a guide to move around - I am completely blind |  |  |  |
| 142. How often do you feel in control of your life? | - Always - Mostly - Sometimes - Only occasionally - Never |  |  |  |
| 143. How much help do you need with jobs around the house (e.g., preparing food, cleaning the house, or gardening)? | - I can do these tasks very quickly and efficiently without any help - I can do these tasks relatively easily without help - I can do these tasks only very slowly without help - I cannot do most of these tasks unless I have help - I can do none of these tasks by myself |  |  |  |
| 144. How often do you feel socially isolated? | - Never - Rarely - Sometimes - Often - Always |  |  |  |
| 145. How well can you hear (using your hearing aid if needed)? | - I have excellent hearing - I hear normally - I have some difficulty hearing, or I do not hear clearly / I have trouble hearing softly spoken people or when there is background noise - I have difficulty hearing things clearly / Often I do not understand what is said / I usually do not take part in conversations because I cannot hear what is said - I hear very little indeed / I cannot fully understand loud voices speaking directly to me - I am completely deaf |  |  |  |
| 146. How often do you feel depressed? | - Never - Almost never - Sometimes - Often - Very often - All the time |  |  |  |
| 147. How happy are you with your close and intimate relationships? | - Very happy - Generally happy - Neither happy nor unhappy - Generally unhappy - Very unhappy |  |  |  |
| 148. How often did you feel in despair over the last seven days? | - Never - Occasionally - Sometimes - Often - All the time |  |  |  |

# CLOSING REFLECTION AND EVALUATION

These next questions ask how you feel about the results of your top surgery.

| **Questions** | **Variables** | **6 wk pre** | **6 wk post** | **1 yr post** |
| --- | --- | --- | --- | --- |
| 149. Looking back on your top surgery, are there any things you would reconsider or do differently? (Select all that apply) | - I would have chosen a different surgeon - I would have had a different surgical procedure - I wish I had surgery earlier in life - I wish I had surgery later in life - The impact on my finances - The positive impact on my relationship/s - The negative impact on my relationship/s - Taking more care during healing process (scar care, limiting movement, etc.) - Having more mental health and/or physical support - I would not have had surgery at all - There is nothing I would change - Prefer not to say - Other/something else: [open text] |  |  |  |
| 150. Please tell us more about this if you want to: | [open text] |  |  |  |
| 151. At this very early stage in the healing process, how satisfied are you with your results? | - Completely satisfied - Very satisfied - Moderately satisfied - Slightly satisfied - Not at all satisfied - Prefer not to say |  |  |  |
| 152. What are you looking forward to in the weeks and months to come now that you have had surgery? | [open text] |  |  |  |
| 153. This is the end of the survey, is there anything else you would like to tell us? | [open text] |  |  |  |

Thank you for completing this questionnaire, we will be in touch with the next follow-up questionnaire in about 11 months.

*If any of the questions we just asked were distressing, we urge you to contact Qlife LGBTI phone and chat counselling and info service (1800 184 527 www.qlife.org.au), Lifeline (13 11 14 www.lifeline.org.au), Suicide Call Back Service (1300 659 467* [*www.suicidecallbackservice.org.au/*](http://www.suicidecallbackservice.org.au/)*), beyondblue (1300 22 4636* [*www.beyondblue.org.au*](http://www.beyondblue.org.au)*), Kids Helpline (1800 55 1800* [*www.kidshelpline.com.au*](http://www.kidshelpline.com.au)*), 13YARN (13 92 76* [*www.13yarn.com.au*](http://www.13yarn.com.au)*), The Butterfly Foundation (1800 33 4673* [*https://butterfly.org.au/*](https://butterfly.org.au/)*) or any other support service.*
