# Supplementary material for: Mental Health, Gender Dysphoria, and Quality of Life in Trans Individuals Following Top Surgery: Protocol for a Prospective Cohort Study
Source: JMIR Res Protoc. 2026 Aug 21;15:e83928. doi: 10.2196/83928 (PMC13496940; doi:10.2196/83928)
Supplement: Supplementary file 3 [file resprot-v15-e83928-s003.docx]

**TOP SURGERY STUDY**

**1 YEAR POST-SURGERY QUESTIONNAIRE**

Welcome to the Top Surgery Study 1-year post-surgery questionnaire. Thank you for consenting to take part in this study.

This study aims to assess the impacts of top surgery (an umbrella term that includes both chest reconstructive surgery and breast augmentation) on the gender dysphoria, mental health, well-being, and quality of life of trans and gender diverse individuals over time. For more information about the research project and the information we want to collect, please refer to the Participant Information Sheet.

If you do not feel able to complete parts of this questionnaire at this time, please feel free to skip any questions you do not feel comfortable answering. You can also choose to withdraw from the study at any point.

We recognise this is a long survey, so please take your time in completing it and come back to it as needed. This questionnaire link is unique to your email address. This means that you can take breaks from the questionnaire rather than having to complete it all at once, provided you access the link using the same device.

This questionnaire link will be active for two weeks from the date we emailed it to you. We will send you a reminder to complete the survey after one week. If you have any questions, please contact us via email at [topsurgery@thekids.org.au](mailto:topsurgery@thekids.org.au).

# DEMOGRAPHICS This first set of questions are about you, so that we can accurately describe everyone involved in this study.

| **Questions** | **Variables** | **6 wk pre** | **6 wk post** | **1 yr post** |
| --- | --- | --- | --- | --- |
| 1. **[mandatory response]** What is your last name?   *We are asking this to link your survey responses to your medical records. Your surgeon will not see your individual responses to this questionnaire. If your last name has changed since your last questionnaire, please let us know your current and previous last names.* | [open text] |  |  |  |
| 2. **[mandatory response]** What is your date of birth?   *We will ask you this on each survey to link your responses.* | - Day/month/year |  |  |  |
| 3. What is your current preferred contact email? | [open text] |  |  |  |
| 4. **[mandatory response]** Would you prefer us to use “chest” or “breast/s” throughout this survey?   *For example, the following question will be phrased as either: “If you experience chest dysphoria, has your chest dysphoria changed since booking surgery?” or “If you experience breast dysphoria, has your breast dysphoria changed since booking surgery?”* | - Chest - Breast/s |  |  |  |
| 5. How do you describe your gender (e.g. woman, man, trans man, brotherboy, bigender, ambigender, demigirl)?  *Feel free to put in more than one identifying term if relevant to you.* | [open text] |  |  |  |
| 6. For the purposes of data analysis we sometimes have to use gender categories. Which of the following terms best describes you: | - Man or male - Woman or female - Non-binary |  |  |  |
| 7. How do you describe your sexuality?   *Feel free to put in more than one identifying term if relevant to you.* | [open text] |  |  |  |
| 8. What best describes your current living situation? (Select all that apply) | - On my own - With a spouse/partner/s - With my parent/s - With other family members - Foster home - Share house - Residential college - Boarding house - Supported accommodation (e.g., Foyer Oxford) - Assisted living/care facility - No fixed accommodation - Prefer not to say - Other, please specify: [open text] |  |  |  |
| 9. What is the postcode where you currently live/spend most of your time? | [open text] |  |  |  |
| 10. What is the highest level of education you have completed? | - Primary school - High school - Technical or trade training/TAFE certificate or diploma - Undergraduate university degree - Postgraduate university degree - Prefer not to say |  |  |  |
| 11. Since your original surgery, have you had any revision surgeries? | - Yes - No - Prefer not to say |  |  |  |
| 12. *If yes*, when did you have your revision surgery/ies?   *Will only display if ‘yes’ is selected in response to having had any revision surgeries.* | [open text] |  |  |  |
| 13. *If no***,** are you planning/or expecting to have a revision surgery?   *Will only display if ‘no’ is selected in response to having had any revision surgeries.* | - Yes, I have a revision surgery booked - Yes, I am expecting to have a revision surgery but have not yet booked it - I might decide to in the future - No, I’m not planning to - Prefer not to say |  |  |  |
| 14. *If yes,* when are you planning or expecting to have revision surgery?   *Will only display if ‘yes’ is selected in response to planning or expecting to have any revision surgeries.* | [open text] |  |  |  |

**GENERAL SUPPORT**

The next few questions ask about the support you’ve received since having your top surgery.

| **Questions** | **Variables** | **6 pre** | **6 Post** | **1 yr** |
| --- | --- | --- | --- | --- |
| 15. If you have a partner/s, how supportive were they during your recovery? | - Very supportive - Somewhat supportive - Neutral - Slightly unsupportive - Not at all supportive - Does not apply to me - Prefer not to say |  |  |  |
| 16. How supportive were your family during your recovery? | - Very supportive - Somewhat supportive - Neutral - Slightly unsupportive - Not at all supportive - Does not apply to me - Prefer not to say |  |  |  |
| 17. How supportive were your friends during your recovery? | - Very supportive - Somewhat supportive - Neutral - Slightly unsupportive - Not at all supportive - Does not apply to me - Prefer not to say |  |  |  |
| 18. Who or what have been the most important sources of support for your mental health following your top surgery? (Select all that apply) | - Immediate family (e.g., siblings, parents) - Extended family (e.g., cousins, grandparents) - Friends/peers - Other trans people - My partner/s - Mental health professional/s - GP - Online groups (e.g., Discord) - Peer support groups (e.g., TransFolk of WA) - Prefer not to say - Other, please specify: [open text] |  |  |  |
| 19. Since having top surgery, if you have accessed mental health support, what types have you accessed? (Select all that apply) | - Peer support (either online or face to face) - Therapy (either online or face to face) - GP or another medical specialist - I have not accessed mental health support - Prefer not to say - Other, please specify [open text] |  |  |  |
| 20. Since having top surgery, what are some of the positive and exciting feelings you have had about your chest/breasts? | [open text] |  |  |  |

**GENDER-Q – HEALTH PROFESSIONAL**

These questions ask about the gender-affirming care you received from your surgeon. Please answer thinking of your most recent interactions. How much do you disagree or agree

that your surgeon:

| **Questions** | **Variables** | **6 wk pre** | **6 wk post** | **1 yr post** |
| --- | --- | --- | --- | --- |
| 181. Treated you with respect? | - Dissatisfied - Somewhat satisfied - Very satisfied - Extremely satisfied |  |  |  |
| 182. Made you feel safe? | - Dissatisfied - Somewhat satisfied - Very satisfied - Extremely satisfied |  |  |  |
| 183. Was trustworthy? | - Dissatisfied - Somewhat satisfied - Very satisfied - Extremely satisfied |  |  |  |
| 184. Made you feel affirmed in your gender identity? | - Dissatisfied - Somewhat satisfied - Very satisfied - Extremely satisfied |  |  |  |
| 185. Asked you questions that were relevant to the treatment you were seeking? | - Dissatisfied - Somewhat satisfied - Very satisfied - Extremely satisfied |  |  |  |
| 186. Helped you access the treatment you need? | - Dissatisfied - Somewhat satisfied - Very satisfied - Extremely satisfied |  |  |  |
| 187. Answered all your questions? | - Dissatisfied - Somewhat satisfied - Very satisfied - Extremely satisfied |  |  |  |
| 188. Involved you in decisions about your treatments? | - Dissatisfied - Somewhat satisfied - Very satisfied - Extremely satisfied |  |  |  |
| 189. Listened to you and understood your concerns? | - Dissatisfied - Somewhat satisfied - Very satisfied - Extremely satisfied |  |  |  |
| 190. Was easy to talk to? | - Dissatisfied - Somewhat satisfied - Very satisfied - Extremely satisfied |  |  |  |
| 191. Helped you have realistic expectations about gender-affirming treatments? | - Dissatisfied - Somewhat satisfied - Very satisfied - Extremely satisfied |  |  |  |
| 192. Provided the highest possible level of care? | - Dissatisfied - Somewhat satisfied - Very satisfied - Extremely satisfied |  |  |  |
| 193. Was thorough? | - Dissatisfied - Somewhat satisfied - Very satisfied - Extremely satisfied |  |  |  |
| 194. Explained treatment options in as much detail as you would like? | - Dissatisfied - Somewhat satisfied - Very satisfied - Extremely satisfied |  |  |  |
| 195. Helped you figure out what was best for you? | - Dissatisfied - Somewhat satisfied - Very satisfied - Extremely satisfied |  |  |  |
| 196. Please tell us more about this if you would like to: | [open text] |  |  |  |

# GENDER-AFFIRMING HORMONES We’re now going to ask some questions about your use of gender-affirming care, including use of hormones and surgeries. By gender-affirming hormones, also known as hormone replacement therapy (HRT), we mean hormones that change your body so that it more closely aligns with your gender identity. By gender-affirming surgeries, we mean procedures that surgically alter your physical appearance to more closely align with your gender identity.

| **Questions** | **Variables** | **6 wk**  **pre** | **6 wk**  **Post** | **1 yr**  **post** |
| --- | --- | --- | --- | --- |
| 21. Are you currently on gender-affirming hormones (e.g., oestrogen, testosterone)? | - Yes - No - Prefer not to say |  |  |  |
| 22. What gender-affirming hormones or similar gender-affirming medications are you currently taking?   (Select all that apply)  *Will only display if ‘yes’ is selected in response to the previous question.* | - Testosterone - Oestrogen - Progesterone - Puberty blockers (e.g., Lucrin) - Anti-androgens (testosterone blockers e.g., Spironolactone or Cyproterone) - Prefer not to say - Other, please specify: [open text] |  |  |  |
| 23. How long have you been on gender-affirming hormones for?   We recognise that some people start and stop gender-affirming hormones so please choose the answer that best represents your experience.   *Will only display if ‘yes’ is selected in response to currently being on gender-affirming hormones.* | - Less than 3 months - 3-6 months - 6-12 months - 1-2 years - 2-5 years - More than 5 years - More than 10 years - Prefer not to say - Other, please specify: [open text] |  |  |  |
| 24. Were you on puberty suppression at any point? | - Yes - No - Prefer not to say |  |  |  |
| 25. Would you have wished to be on puberty suppression at some point but were unable to access it?   *Will only display if ‘no’ is selected in response to the previous question.* | - Yes - No - Prefer not to say |  |  |  |
| 26. If you would you like to include further information about your experiences with gender-affirming hormones (including puberty blockers) in relation to top surgery, please do so here: | [open text] |  |  |  |
| 27. Is top surgery the only gender-affirming surgery you have had? | - Yes - No - Prefer not to say |  |  |  |
| 28. Have you had any of the following gender-affirming surgeries?   *Will only display if ‘no’ is selected in response to the previous question.* | - Facial surgery - Vocal surgery - Metoidioplasty (creation of a penis or phallus from a hormonally enlarged clitoris) - Phalloplasty (a number of procedures that surgically creates a penis or phallus from existing tissue) - Vaginoplasty (creation of a vagina and vulva from existing tissue) - Vulvoplasty (creation of a vulva from existing tissue with no vagina or a shallow depth vagina) - Hysterectomy (removal of all or some parts of the uterus, cervix, fallopian tubes, and ovaries) - Oophorectomy (removal of one or both ovaries) - Orchiectomy (removal of testes) - Prefer not to say - Another type, please specify [open text] |  |  |  |

**MENTAL HEALTH**

We would like to ask you about your mental health and wellbeing. This includes questions about suicide and self-harm. We understand that these experiences can be common. This research and your responses are confidential. If you are in a high-risk situation, we will attempt to contact you to assist you in getting support. We will not share your responses with anyone else or contact anyone on your behalf without your consent.

*If any of the following questions upset you, we urge you to contact Qlife LGBTI phone and chat counselling and info service (1800 184 527 www.qlife.org.au), Lifeline (13 11 14 www.lifeline.org.au), Suicide Call Back Service (1300 659 467* [*www.suicidecallbackservice.org.au/*](http://www.suicidecallbackservice.org.au/)*), beyondblue (1300 22 4636* [*www.beyondblue.org.au*](http://www.beyondblue.org.au)*), Kids Helpline (1800 55 1800* [*www.kidshelpline.com.au*](http://www.kidshelpline.com.au)*), 13YARN (13 92 76* [*www.13yarn.com.au*](http://www.13yarn.com.au)*) or any other support service.*

**ANXIETY – GAD-7**

Over the past 2 weeks, how often have you been bothered by any of the following problems?

| **Questions** | **Variables** | **6 wk pre** | **6 wk post** | **1 yr post** |
| --- | --- | --- | --- | --- |
| 29. Feeling nervous, anxious or on edge | - Not at all - Several days - More than half the days - Nearly every day |  |  |  |
| 30. Not being able to stop or control worrying | - Not at all - Several days - More than half the days - Nearly every day |  |  |  |
| 31. Worrying too much about different things | - Not at all - Several days - More than half the days - Nearly every day |  |  |  |
| 32. Trouble relaxing | - Not at all - Several days - More than half the days - Nearly every day |  |  |  |
| 33. Being so restless that it is hard to sit still | - Not at all - Several days - More than half the days - Nearly every day |  |  |  |
| 34. Becoming easily annoyed or irritable | - Not at all - Several days - More than half the days - Nearly every day |  |  |  |
| 35. Feeling afraid as if something awful might happen | - Not at all - Several days - More than half the days - Nearly every day |  |  |  |
| 36. If you checked off any problems, how difficult have these problems made it for you to do your work, take care of things at home, or get along with other people? | - Not difficult at all - Somewhat difficult - Very difficult - Extremely difficult |  |  |  |

**DEPRESSION – PHQ-9**

Over the past 2 weeks, how often have you been bothered by any of the following problems?

| **Questions** | **Responses** | **6 wk pre** | **6 wk post** | **1 yr post** |
| --- | --- | --- | --- | --- |
| 37. Little interest or pleasure in doing things | - Not at all - Several days - More than half the days - Nearly every day |  |  |  |
| 38. Feeling down, depressed, or hopeless | - Not at all - Several days - More than half the days - Nearly every day |  |  |  |
| 39. Trouble falling or staying asleep, or sleeping too much | - Not at all - Several days - More than half the days - Nearly every day |  |  |  |
| 40. Feeling tired or having little energy | - Not at all - Several days - More than half the days - Nearly every day |  |  |  |
| 41. Poor appetite or overeating | - Not at all - Several days - More than half the days - Nearly every day |  |  |  |
| 42. Feeling bad about yourself or that you are a failure or have let yourself or your family down | - Not at all - Several days - More than half the days - Nearly every day |  |  |  |
| 43. Trouble concentrating on things, such as reading the newspaper or watching television | - Not at all - Several days - More than half the days - Nearly every day |  |  |  |
| 44. Moving or speaking so slowly that other people could have noticed / Or the opposite, being so fidgety or restless that you have been moving around a lot more than usual | - Not at all - Several days - More than half the days - Nearly every day |  |  |  |
| 45. Thoughts that you would be better off dead, or of hurting yourself | - Not at all - Several days - More than half the days - Nearly every day |  |  |  |
| 46. If you checked off any problems, how difficult have these problems made it for your to do your work, take care of things at home, or get along with other people? | - Not difficult at all - Somewhat difficult - Very difficult - Extremely difficult |  |  |  |

**SELF-HARM AND SUICIDE**

| **Questions** | **Variables** | **6 wk pre** | **6 wk post** | **1 yr post** |
| --- | --- | --- | --- | --- |
| 47. Within the last 12 months have you wanted to harm yourself (without wanting to end your life)? | - Yes - No - Prefer not to say |  |  |  |
| 48. Within the last 12 months have you harmed yourself (without wanting to end your life)? | - Yes - No - Prefer not to say |  |  |  |
| 49. Do you use non-suicidal self-harm to cope with gender-related concerns? | - Yes - No - Prefer not to say |  |  |  |
| 50. Do you use non-suicidal self-harm to cope with chest/breast dysphoria?   1. *Will only display if ‘yes’ is selected in response to the previous question.* | - Yes - No - Prefer not to say |  |  |  |
| 51. Within the last 12 months have you had serious thoughts about ending your life? | - Yes - No - Prefer not to say |  |  |  |
| 52. Within the last 12 months have you tried to kill yourself or made a suicide attempt? | - Yes - No - Prefer not to say |  |  |  |
| 53. Within the last 12 months have you deliberately done something reckless to purposely risk your life? | - Yes - No - Prefer not to say |  |  |  |

*If any of the previous questions caused you distress, please contact Qlife LGBTI phone and chat counselling and info service (1800 184 527 www.qlife.org.au), Lifeline (13 11 14 www.lifeline.org.au), Suicide Call Back Service (1300 659 467* [*www.suicidecallbackservice.org.au/*](http://www.suicidecallbackservice.org.au/)*), beyondblue (1300 22 4636* [*www.beyondblue.org.au*](http://www.beyondblue.org.au)*), Kids Helpline (1800 55 1800* [*www.kidshelpline.com.au*](http://www.kidshelpline.com.au)*), 13YARN (13 92 76* [*www.13yarn.com.au*](http://www.13yarn.com.au)*) or any other support service.*

**DIAGNOSES**

The next questions ask about any diagnoses you may have currently or have received in the past.

Have you been diagnosed by a doctor, psychologist, psychiatrist, or other medical professional with any of the following? Some of these diagnoses may have occurred in the past as well as currently – if this is the case, please select both “currently diagnosed” and “ever diagnosed”. Please tick all that apply.

| **Questions** | **Variables** | **6 wk pre** | | **6 wk post** | **1 yr post** |
| --- | --- | --- | --- | --- | --- |
| 54. Depression (e.g., major depressive disorder, persistent depressive disorder, post-partum depression) | - Currently diagnosed - Ever diagnosed - Not diagnosed - Prefer not to say |  |  | |  |
| 55. An anxiety disorder (e.g., generalised anxiety disorder, social anxiety, panic disorder) | - Currently diagnosed - Ever diagnosed - Not diagnosed - Prefer not to say |  |  | |  |
| 56. Post-traumatic stress disorder (PTSD) | - Currently diagnosed - Ever diagnosed - Not diagnosed - Prefer not to say |  |  | |  |
| 57. Obsessive compulsive disorder (OCD) | - Currently diagnosed - Ever diagnosed - Not diagnosed - Prefer not to say |  |  | |  |
| 58. Bipolar disorder | - Currently diagnosed - Ever diagnosed - Not diagnosed - Prefer not to say |  |  | |  |
| 59. A personality disorder | - Currently diagnosed - Ever diagnosed - Not diagnosed - Prefer not to say |  |  | |  |
| 60. Psychosis (e.g., schizophrenia, post-partum psychosis, drug-induced psychosis) | - Currently diagnosed - Ever diagnosed - Not diagnosed - Prefer not to say |  |  | |  |
| 61. An eating disorder | - Currently diagnosed - Ever diagnosed - Not diagnosed - Prefer not to say |  |  | |  |
| 62. Substance use disorder | - Currently diagnosed - Ever diagnosed - Not diagnosed - Prefer not to say |  |  | |  |
| 63. Autism | - Currently diagnosed - Ever diagnosed - Not diagnosed - Prefer not to say |  |  | |  |
| 64. Attention-deficit/hyperactivity disorder (ADHD) | - Currently diagnosed - Ever diagnosed - Not diagnosed - Prefer not to say |  |  | |  |
| 65. Please tell us more about your mental health, including any additional diagnoses, here if you wish to: | [open text] |  |  | |  |
| 66. Do you feel your mental health has changed (positively or negatively) since having surgery? If so, please tell us how: | [open text] |  |  | |  |
| 67. Do you have any disabilities that have impacted your experience of recovering from surgery? | - Yes - No - Prefer not to say |  |  | |  |
| 68. Do you have any chronic health conditions that have impacted your experience of recovering from surgery? | - Yes - No - Prefer not to say |  |  | |  |
| 69. Do you identify as being neurodivergent (with or without a formal diagnosis of autism, ADHD and/or a related condition)? | - Yes - No - Prefer not to say |  |  | |  |
| 70. *If yes to any of the above questions,* please tell us more about these experiences if you wish to:   *Will only display if ‘yes’ is selected in response to question 67 or 68 or 69.* | [open text] |  |  | |  |

# GENDER EUPHORIA SCALE (GES)

Gender euphoria is a positive feeling about your gender identity, gender expression, or gender affirmation. Gender euphoria may involve feeling happy, confident, content, safe, or another feeling you recognise as being positive to you. Gender euphoria may arise when you feel good about your gender, when your gender is accepted and affirmed by yourself or others, or when you feel true to yourself and your gender.

The next series of questions ask about your experiences of gender euphoria. On a scale from 1 – 5, where 1 = no gender euphoria and 5 = very strong gender euphoria, how strong has your gender euphoria felt in the following scenarios over the past two weeks:

| **Questions** | **Responses** | **6 wk pre** | **6 wk post** | **1 yr post** |
| --- | --- | --- | --- | --- |
| 71. When people use my   correct pronouns | - No gender euphoria - Slight gender euphoria - Moderate gender euphoria - Strong gender euphoria - Very strong gender euphoria - This scenario does not apply to me (NA) |  |  |  |
| 72. When I look in the mirror | - No gender euphoria - Slight gender euphoria - Moderate gender euphoria - Strong gender euphoria - Very strong gender euphoria - This scenario does not apply to me (NA) |  |  |  |
| 73. When my gender is   affirmed in everyday encounters (e.g., at shops, restaurants) | - No gender euphoria - Slight gender euphoria - Moderate gender euphoria - Strong gender euphoria - Very strong gender euphoria - This scenario does not apply to me (NA) |  |  |  |
| 74. When I see myself represented in the workplace | - No gender euphoria - Slight gender euphoria - Moderate gender euphoria - Strong gender euphoria - Very strong gender euphoria - This scenario does not apply to me (NA) |  |  |  |
| 75. When people treat me the way I want to be treated, in relation to my gender | - No gender euphoria - Slight gender euphoria - Moderate gender euphoria - Strong gender euphoria - Very strong gender euphoria - This scenario does not apply to me (NA) |  |  |  |
| 76. When my gender is affirmed at work | - No gender euphoria - Slight gender euphoria - Moderate gender euphoria - Strong gender euphoria - Very strong gender euphoria - This scenario does not apply to me (NA) |  |  |  |
| 77. When I see my face in the mirror | - No gender euphoria - Slight gender euphoria - Moderate gender euphoria - Strong gender euphoria - Very strong gender euphoria - This scenario does not apply to me (NA) |  |  |  |
| 78. When I see transgender, gender diverse, or non-binary people represented in the media | - No gender euphoria - Slight gender euphoria - Moderate gender euphoria - Strong gender euphoria - Very strong gender euphoria - This scenario does not apply to me (NA) |  |  |  |
| 79. When people use the correct language to describe my gender | - No gender euphoria - Slight gender euphoria - Moderate gender euphoria - Strong gender euphoria - Very strong gender euphoria - This scenario does not apply to me (NA) |  |  |  |
| 80. When I connect with other transgender, gender diverse, or non-binary people online | - No gender euphoria - Slight gender euphoria - Moderate gender euphoria - Strong gender euphoria - Very strong gender euphoria - This scenario does not apply to me (NA) |  |  |  |
| 81. When I see people who experience their gender in a similar way to me | - No gender euphoria - Slight gender euphoria - Moderate gender euphoria - Strong gender euphoria - Very strong gender euphoria - This scenario does not apply to me (NA) |  |  |  |
| 82. When strangers affirm my gender in public | - No gender euphoria - Slight gender euphoria - Moderate gender euphoria - Strong gender euphoria - Very strong gender euphoria - This scenario does not apply to me (NA) |  |  |  |
| 83. When I see my facial hair (or lack thereof) | - No gender euphoria - Slight gender euphoria - Moderate gender euphoria - Strong gender euphoria - Very strong gender euphoria - This scenario does not apply to me (NA) |  |  |  |
| 84. When my gender is affirmed in education settings | - No gender euphoria - Slight gender euphoria - Moderate gender euphoria - Strong gender euphoria - Very strong gender euphoria - This scenario does not apply to me (NA) |  |  |  |
| 85. When my gender is legally recognised | - No gender euphoria - Slight gender euphoria - Moderate gender euphoria - Strong gender euphoria - Very strong gender euphoria - This scenario does not apply to me (NA) |  |  |  |
| 86. When I feel connected to the transgender, gender diverse, and non-binary community | - No gender euphoria - Slight gender euphoria - Moderate gender euphoria - Strong gender euphoria - Very strong gender euphoria - This scenario does not apply to me (NA) |  |  |  |
| 87. When I access spaces that affirm my gender (e.g., my workplace, parties) | - No gender euphoria - Slight gender euphoria - Moderate gender euphoria - Strong gender euphoria - Very strong gender euphoria - This scenario does not apply to me (NA) |  |  |  |
| 88. When I see myself represented in the community | - No gender euphoria - Slight gender euphoria - Moderate gender euphoria - Strong gender euphoria - Very strong gender euphoria - This scenario does not apply to me (NA) |  |  |  |
| 89. When I see my body in the mirror | - No gender euphoria - Slight gender euphoria - Moderate gender euphoria - Strong gender euphoria - Very strong gender euphoria - This scenario does not apply to me (NA) |  |  |  |
| 90. When I am welcomed into spaces designated for my gender | - No gender euphoria - Slight gender euphoria - Moderate gender euphoria - Strong gender euphoria - Very strong gender euphoria - This scenario does not apply to me (NA) |  |  |  |
| 91. When my gender is affirmed by my family of origin (e.g., parents, guardians, siblings) | - No gender euphoria - Slight gender euphoria - Moderate gender euphoria - Strong gender euphoria - Very strong gender euphoria - This scenario does not apply to me (NA) |  |  |  |
| 92. When I am included by others who have the same gender as me | - No gender euphoria - Slight gender euphoria - Moderate gender euphoria - Strong gender euphoria - Very strong gender euphoria - This scenario does not apply to me (NA) |  |  |  |
| 93. When my gender is affirmed by strangers | - No gender euphoria - Slight gender euphoria - Moderate gender euphoria - Strong gender euphoria - Very strong gender euphoria - This scenario does not apply to me (NA) |  |  |  |
| 94. When I discuss my gender in online forums (e.g., Reddit, Facebook groups) | - No gender euphoria - Slight gender euphoria - Moderate gender euphoria - Strong gender euphoria - Very strong gender euphoria - This scenario does not apply to me (NA) |  |  |  |
| 95. When I connect with other transgender, gender diverse, or non-binary people in person | - No gender euphoria - Slight gender euphoria - Moderate gender euphoria - Strong gender euphoria - Very strong gender euphoria - This scenario does not apply to me (NA) |  |  |  |
| 96. When my identity documents (e.g., passport, driver’s license) accurately reflect my gender | - No gender euphoria - Slight gender euphoria - Moderate gender euphoria - Strong gender euphoria - Very strong gender euphoria - This scenario does not apply to me (NA) |  |  |  |

# CHEST/BREAST DYSPHORIA - OLSON-KENNEDY SCALE

Some trans and gender diverse people experience dysphoria. Dysphoria can be described as the distress experienced when your sex presumed at birth and gender identity do not align. Dysphoria may be associated with certain parts of the body, cultural expectations of gender and/or how others perceive you.

The following statements ask you to consider your feelings in the last two weeks.

| **Questions** | **Variables** | **6 wk pre** | **6 wk post** | **1 yr post** |
| --- | --- | --- | --- | --- |
| 97. I like looking at my chest/breasts in the mirror   *(Original question: I like looking at my chest in the mirror. The option “Prefer not to say” has been added.)* | - Never - Sometimes - Frequently - All the time - Prefer not to say |  |  |  |
| 98. Taking a bath/shower is difficult because I have to see my chest/breasts   *(Original question: Taking a shower/bath is difficult because I have to see my chest. The option “Prefer not to say” has been added.)* | - Never - Sometimes - Frequently - All the time - Prefer not to say |  |  |  |
| 99. I avoid going to the beach and/or swimming in public places because of my chest/breasts   *(Original question: I avoid going to the beach and/or swimming in public places because of my chest. The option “Prefer not to say” has been added.)* | - Never - Sometimes - Frequently - All the time - Prefer not to say |  |  |  |
| 100. I get misgendered because of my chest/breasts   *(Original question: I get gendered as female because of my chest. The option “Prefer not to say” has been added.)* | - Never - Sometimes - Frequently - All the time - Prefer not to say |  |  |  |
| 101. If you are currently dating or interested in dating:  Dating/forming intimate partner relationships is difficult because of my chest/breasts   *(Original question: Dating/forming intimate partnerships is more difficult because of my chest. The option “Prefer not to say” has been added.)* | - Never - Sometimes - Frequently - All the time - Does not apply to me - Prefer not to say |  |  |  |
| 102. Physical intimacy/sexual activity is difficult because of my chest/breasts   *(Original question: Physical intimacy/sexual activity is difficult because of my chest. The option “does not apply to me” was added to be inclusive of asexual people. The option “Prefer not to say” has also been added.)* | - Never - Sometimes - Frequently - All the time - Does not apply to me - Prefer not to say |  |  |  |
| 103. I have struggled to make future plans because of my chest/breasts   *(Original question: I have struggled to make future plans because of my chest. The option “Prefer not to say” has been added.)* | - Never - Sometimes - Frequently - All the time - Prefer not to say |  |  |  |
| 104. I avoid exercise because of my chest/breasts   *(Original question: I avoid exercise because of my chest. The option “Prefer not to say” has been added.)* | - Never - Sometimes - Frequently - All the time - Prefer not to say |  |  |  |
| 105. I avoid shopping/buying clothing because of my chest/breasts   *(Original question: I avoid shopping/buying clothing because of my chest. The option “Prefer not to say” has been added.)* | - Never - Sometimes - Frequently - All the time - Prefer not to say |  |  |  |
| 106. I avoid seeking medical care because of my chest/breasts   *(Original question: I avoid seeking medical care because of my chest. The option “Prefer not to say” has been added.)* | - Never - Sometimes - Frequently - All the time - Prefer not to say |  |  |  |
| 107. I feel like my life hasn’t started because of my chest/breasts   *(Original question: I feel like my life hasn’t started because of my chest. The option “Prefer not to say” has been added.)* | - Never - Sometimes - Frequently - All the time - Prefer not to say |  |  |  |
| 108. I avoid swimming in private places because of my chest/breasts   *(Original question: I avoid swimming in private places because of my chest. The option “Prefer not to say” has been added.)* | - Never - Sometimes - Frequently - All the time - Prefer not to say |  |  |  |
| 109. I have to buy/wear certain clothes because of my chest/breasts   *(Original question: I have to buy/wear certain clothes because of my chest. The option “Prefer not to say” has been added.)* | - Never - Sometimes - Frequently - All the time - Prefer not to say |  |  |  |
| 110. I sleep with a binder/breast forms or bra on at night   *(Original question: I sleep with a binder on at night. The option “Prefer not to say” has been added.)* | - Never - Sometimes - Frequently - All the time - Prefer not to say |  |  |  |
| 111. I avoid using change rooms because of my chest/breasts   *(Original question: I avoid using locker rooms because of my chest. The option “Prefer not to say” has been added.)* | - Never - Sometimes - Frequently - All the time - Prefer not to say |  |  |  |
| 112. I worry that people are looking at my chest/breasts   *(Original question: I worry that people are looking at my chest. The option “Prefer not to say” has been added.)* | - Never - Sometimes - Frequently - All the time - Prefer not to say |  |  |  |
| 113. I participate in life less than others because of my chest/breasts   *(Original question: I participate in life less than others because of my chest. The option “Prefer not to say” has been added.)* | - Never - Sometimes - Frequently - All the time - Prefer not to say |  |  |  |
| 114. I feel connected to/at ease with my chest/breasts | - Never - Sometimes - Frequently - All the time - Prefer not to say |  |  |  |
| 115. In the last 3 weeks, I have enjoyed expressing my gender through clothing   (*The option “Prefer not to say” has been added.)* | - Never - Sometimes - Frequently - All the time - Prefer not to say |  |  |  |
| 116. I like looking at my chest/breasts with clothing on   (*The option “Prefer not to say” has been added.)* | - Never - Sometimes - Frequently - All the time - Prefer not to say |  |  |  |
| 117. I feel confident in my body   (*The option “Prefer not to say” has been added.)* | - Never - Sometimes - Frequently - All the time - Prefer not to say |  |  |  |

# DYSPHORIA - **ADDITIONAL QUESTIONS**

| **Questions** | **Variables** | **6 wk pre** | **6 wk post** | **1 yr post** |
| --- | --- | --- | --- | --- |
| 118. How often have you experienced chest/breast dysphoria within the last two weeks? | - Never - Rarely - Sometimes - Often - Always - Prefer not to say |  |  |  |
| 119. *If you experience chest /breast dysphoria*, has your chest/breast dysphoria changed since having surgery?   *Will only display if something other than ‘never’* ***or*** *‘prefer not to say’ is selected in response to the previous question.* | - My chest/breast dysphoria has increased since having surgery - My chest/breast dysphoria has decreased since having surgery - My chest/breast dysphoria has stayed the same since having surgery - Does not apply to me - Prefer not to say - Other, please specify: [open text] |  |  |  |
| 120. *If you are currently on gender-affirming hormones,* what has impacted your chest/breast dysphoria more? | - Hormones - Surgery - Hormones and surgery have had an equal impact - Not sure - Does not apply to me - Prefer not to say |  |  |  |
| 121. Is there anything else you would like to tell us about your experience of chest/breast dysphoria? | [open text] |  |  |  |
| 122. Do you currently experience dysphoria in other parts of your body (other than chest/breasts)?  *By currently we are asking about the last two weeks.* | - Never - Rarely - Sometimes - Often - Always - Prefer not to say |  |  |  |
| 123. *If you currently experience dysphoria,* has your dysphoria increased in other parts of your body since surgery?   *Will only display if something other than ‘never’ or ‘prefer not to say’ is selected in response to the previous question.* | - I’m experiencing more dysphoria in other parts of my body - I’m experiencing dysphoria in parts of my body I wasn’t before - Does not apply to me - Prefer not to say |  |  |  |
| 124. If there is anything else you would like to tell us about your experience of dysphoria please do so here: | [open text] |  |  |  |

**EXERCISE AND DIET**

**IPAQ**

We are interested in finding out about the kinds of physical activities that people do as part of their everyday lives. The questions will ask you about the time you spent being physically active in the last 7 days. Please answer each question even if you do not consider yourself to be an active person. Please think about the activities you do at work, as part of your house and yard work, to get from place to place, and in your spare time for recreation, exercise or sport.

| **Questions** | **Responses** | **6 wk pre** | **6 wk post** | **1 yr post** |
| --- | --- | --- | --- | --- |
| 125. Thinking about the **vigorous** physical activities that you did in the last 7 days. **Vigorous** physical activities refer to activities that take hard physical effort and make you breathe much harder than normal. Think only about those physical activities that you did for at least 10 minutes at a time.  During the last 7 days, on how many days did you do **vigorous** physical activities like heavy lifting, digging, aerobics, or fast bicycling? | - Days per week: [open text] - No vigorous physical activities |  |  |  |
| 126. How much time did you usually spend doing **vigorous** physical activities on one of those days?   *Will only display if any days are selected in response to vigorous physical activity.* | - Hours per day: [open text] - Minutes per day: [open text] - Don’t know/Not sure |  |  |  |
| 127. Think about all the **moderate** activities that you did in the last 7 days. **Moderate** activities refer to activities that take moderate physical effort and make you breathe somewhat harder than normal. Think only about those physical activities that you did for at least 10 minutes at a time.  During the last 7 days, on how many days did you do **moderate** physical activities like carrying light loads, bicycling at a regular pace, or doubles tennis? Do not include walking. | - Days per week: [open text] - No moderate physical activities |  |  |  |
| 128. How much time did you usually spend doing **moderate** physical activities on one of those days?   *Will only display if any days are selected in response to moderate physical activity.* | - Hours per day: [open text] - Minutes per day: [open text] - Don’t know/Not sure |  |  |  |
| 129. Think about the time you spent **walking** in the last 7 days. This includes at work and at home, walking to travel from place to place, and any other walking that you have done solely for recreation, sport, exercise, or leisure.  During the last 7 days, on how many days did you **walk** for at least 10 minutes at a time? | - Days per week: [open text] - No walking |  |  |  |
| 130. How much time did you usually spend **walking** on one of those days?   *Will only display if any days are selected in response to walking.* | - Hours per day: [open text] - Minutes per day: [open text] - Don’t know/not sure |  |  |  |
| 131. The last question is about the time you spent **sitting** on weekdays during the last 7 days. Include time spent at work, at home, while doing course work and during leisure time. This may include time spent sitting at a desk, visiting friends, reading, or sitting or lying down to watch television.  During the last 7 days, how much time did you spend **sitting** on a **weekday**? | - Hours per day: [open text] - Minutes per day: [open text] - Don’t know/Not sure |  |  |  |

# MASC/FEM EXERCISE AND EATING

The next few questions ask about changing your body to look more masculine/feminine through eating and exercise.

*If any of the following questions cause you distress, The Butterfly Foundation has a free national helpline for anyone wanting support for eating disorders or body image concerns (1800 33 4673 https://butterfly.org.au/).*

| **Questions** | **Responses** | | **6 wk pre** | **6 wk post** | **1 yr post** |
| --- | --- | --- | --- | --- | --- |
| 132. Do you limit how much you eat because of gender-related concerns? | - Never - Sometimes - Often - Very often - Prefer not to say | |  |  |  |
| 133. Do you increase how much you eat because of gender-related concerns? | - Never - Sometimes - Often - Very often - Prefer not to say |  | |  |  |
| 134. Do you limit how much you exercise because of gender-related concerns? | - Never - Sometimes - Often - Very often - Prefer not to say |  | |  |  |
| 135. Do you increase how much you exercise because of gender-related concerns? | - Never - Sometimes - Often - Very often - Prefer not to say |  | |  |  |
| 136. How often would you like to exercise but can’t for a particular reason? (e.g., because it is expensive, because you are uncomfortable leaving home, etc.) | - Never - Sometimes - Often - Very often - Prefer not to say |  | |  |  |
| 137. When considering exercise, please describe any challenges you face that relate to your breasts/chest: | [open text] |  | |  |  |
| 138. Please tell us more about any of your answers above if you wish to: | [open text] |  | |  |  |

**DISORDERED EATING**

The next few questions ask about feelings and/or behaviours related to disordered eating.

On how many of the past 7 days...

| **Questions** | **Variables** | **6 wk pre** | **6 wk post** | **1 yr post** |
| --- | --- | --- | --- | --- |
| 139. Have you been deliberately trying to limit the amount of food you eat to influence your weight or shape (whether or not you have succeeded)? | - 0 days - 1-2 days - 3-5 days - 6-7 days |  |  |  |
| 140. Have you gone for long periods of time (e.g., 8 or more waking hours) without eating anything at all in order to influence your weight or shape? | - 0 days - 1-2 days - 3-5 days - 6-7 days |  |  |  |
| 141. Has thinking about food, eating or calories made it very difficult to concentrate on things you are interested in (such as working, following a conversation or reading)? | - 0 days - 1-2 days - 3-5 days - 6-7 days |  |  |  |
| 142. Has thinking about your weight or shape made it very difficult to concentrate on things you are interested in (such as working, following a conversation or reading)? | - 0 days - 1-2 days - 3-5 days - 6-7 days |  |  |  |
| 143. Have you had a definite fear that you might gain weight? | - 0 days - 1-2 days - 3-5 days - 6-7 days |  |  |  |
| 144. Have you had a strong desire to lose weight? | - 0 days - 1-2 days - 3-5 days - 6-7 days |  |  |  |
| 145. Have you tried to control your weight or shape by making yourself sick (vomit) or taking laxatives? | - 0 days - 1-2 days - 3-5 days - 6-7 days |  |  |  |
| 146. Have you exercised in a driven or compulsive way as a means of controlling your weight, shape or body fat, or to burn off calories? | - 0 days - 1-2 days - 3-5 days - 6-7 days |  |  |  |
| 147. Have you had a sense of having lost control over your eating (at the time that you were eating)? | - 0 days - 1-2 days - 3-5 days - 6-7 days |  |  |  |
| 148. On how many of these days (i.e. days on which you had a sense of having lost control over your eating) did you eat what other people would regard as an unusually large amount of food in one go? | - 0 days - 1-2 days - 3-5 days - 6-7 days |  |  |  |

Over the past 7 days...

| **Questions** | **Variables** | **6 wk pre** | **6 wk post** | **1 yr post** |
| --- | --- | --- | --- | --- |
| 149. Has your weight or shape influenced how you think about (judge) yourself as a person? | - Not at all - Slightly - Moderately - Markedly |  |  |  |
| 150. How dissatisfied have you been with your weight or shape? | - Not at all - Slightly - Moderately - Markedly |  |  |  |

If any of the previous questions caused you distress, please contact the The Butterfly Foundation on [*1800 33 4673*](tel:1800%2033%204673) or at [*https://butterfly.org.au/*](https://butterfly.org.au/).

**QUALITY OF LIFE – AQoL-8D**

The following questions are about your general health and quality of life. These questions are from a measure used to look at quality of life. This information gives us insight into how top surgery impacts trans people’s day-to-day quality of life over time.

Select the option that best describes your situation over the last week.

| **Questions** | **Variables** | **6 wk pre** | **6 wk post** | **1 yr post** |
| --- | --- | --- | --- | --- |
| 151. How much energy do you have to do the things you want to do? I am: | - Always full of energy - Usually full of energy - Occasionally energetic - Usually tired and lacking energy - Always tired and lacking energy |  |  |  |
| 152. How often do you feel socially excluded or left out? | - Never - Rarely - Sometimes - Often - Always |  |  |  |
| 153. How easy or difficult is it for you to get around by yourself outside your place of residence (e.g., shopping, visiting)? | - Getting around is enjoyable and easy - I have no difficulty getting around outside my house - A little difficulty - Moderate difficulty - A lot of difficulty - I cannot get around unless somebody is there to help me |  |  |  |
| 154. Does your health affect your role in your community (e.g., residential, sporting, church, or cultural activities)? | - My role in the community is unaffected by my health - There are some parts of my community role I cannot carry out - There are many parts of my community role I cannot carry out - I cannot carry out any part of my community role |  |  |  |
| 155. How often do you feel sad? | - Never - Rarely - Some of the time - Usually - Nearly all the time |  |  |  |
| 156. How often do you experience serious pain? I experience it: | - Very rarely - Less than once a week - Three to four times a week - Most of the time |  |  |  |
| 157. How much confidence do you have in yourself? | - Complete confidence - A lot - A moderate amount - A little - None at all |  |  |  |
| 158. Do you normally feel calm and tranquil or agitated? I am: | - Always calm and tranquil - Usually calm and tranquil - Sometimes calm and tranquil, sometimes agitated - Usually agitated - Always agitated |  |  |  |
| 159. Does your health affect your relationship with your family? | - My role in the family is unaffected by my health - There are some parts of my family role I cannot carry out - There are many parts of my family role I cannot carry out - I cannot carry out any part of my family role |  |  |  |
| 160. How satisfying are your close relationships (family and friends)? | - Very satisfying - Satisfying - Neither satisfying nor dissatisfying - Dissatisfying - Unpleasant - Very unpleasant |  |  |  |
| 161. How well do you communicate with others (talking, signing, texting, being understood by others and understanding them)? | - I have no trouble speaking to them or understanding what they are saying - I have some difficulty being understood by people who do not know me / I have no trouble understanding what others are saying to me - I am understood only by people who know me well / I have great trouble understanding what others are saying to me - I cannot adequately communicate with others |  |  |  |
| 162. How often do you have trouble sleeping? | - Never - Almost never - Sometimes - Often - All the time |  |  |  |
| 163. How often do you feel worthless? | - Never - Almost never - Sometimes - Usually - Always |  |  |  |
| 164. How often do you feel angry? | - Never - Almost never - Sometimes - Often - All the time |  |  |  |
| 165. How easy or difficult is it for you to move around (using any aids or equipment you need e.g., a wheelchair, frame, or stick)? | - I am very mobile - I have no difficulty with mobility - I have some difficulty with mobility (for example, going uphill) - I have difficulty with mobility / I can go short distance only - I have a lot of difficulty with mobility / I need someone to help me - I am bedridden |  |  |  |
| 166. Do you ever feel like hurting yourself? | - Never - Almost never - Sometimes - Often - All the time |  |  |  |
| 167. How enthusiastic do you feel? | - Extremely - Very - Somewhat - Not much - Not at all |  |  |  |
| 168. How often did you feel worried in the last seven days? | - Never - Occasionally - Sometimes - Often - All the time |  |  |  |
| 169. How difficult is it for you to wash, toilet, dress yourself, eat or care for your appearance? | - These tasks are very easy for me - I have no real difficulty in carrying out these tasks - I find some of these tasks difficult, but I manage to do them on my own - Many of these tasks are difficult, and I need help to do them - I cannot do these tasks by myself at all |  |  |  |
| 170. How often do you feel happy? | - All the time - Mostly - Sometimes - Almost never - Never |  |  |  |
| 171. How much do you feel you can cope with life’s problems? | - Completely - Mostly - Partly - Very little - Not at all |  |  |  |
| 172. How much pain or discomfort do you experience? | - None at all - I have moderate pain - I suffer from severe pain - I suffer unbearable pain |  |  |  |
| 173. How much do you enjoy your close relationships (family and friends)? | - Immensely - A lot - A little - Not much - I hate it |  |  |  |
| 174. How often does pain interfere with your usual activities? | - Never - Rarely - Sometimes - Often - Always |  |  |  |
| 175. How often do you feel pleasure? | - Always - Usually - Sometimes - Almost never - Never |  |  |  |
| 176. How much of a burden do you feel you are to other people? | - Not at all - A little - A moderate amount - A lot - Totally |  |  |  |
| 177. How content are you with your life? | - Extremely - Mainly - Moderately - Slightly - Not at all |  |  |  |
| 178. How well can you see (using your glasses or contact lenses if they are needed)? | - I have excellent sight - I see normally - I have some difficulty focusing on things, or I do not see them sharply e.g., small print, a newspaper or seeing objects in the distance - I have a lot of difficulty seeing things / My vision is blurred / I can see just enough to get by with - I only see general shapes / I need a guide to move around - I am completely blind |  |  |  |
| 179. How often do you feel in control of your life? | - Always - Mostly - Sometimes - Only occasionally - Never |  |  |  |
| 180. How much help do you need with jobs around the house (e.g., preparing food, cleaning the house, or gardening)? | - I can do these tasks very quickly and efficiently without any help - I can do these tasks relatively easily without help - I can do these tasks only very slowly without help - I cannot do most of these tasks unless I have help - I can do none of these tasks by myself |  |  |  |
| 181. How often do you feel socially isolated? | - Never - Rarely - Sometimes - Often - Always |  |  |  |
| 182. How well can you hear (using your hearing aid if needed)? | - I have excellent hearing - I hear normally - I have some difficulty hearing, or I do not hear clearly / I have trouble hearing softly spoken people or when there is background noise - I have difficulty hearing things clearly / Often I do not understand what is said / I usually do not take part in conversations because I cannot hear what is said - I hear very little indeed / I cannot fully understand loud voices speaking directly to me - I am completely deaf |  |  |  |
| 183. How often do you feel depressed? | - Never - Almost never - Sometimes - Often - Very often - All the time |  |  |  |
| 184. How happy are you with your close and intimate relationships? | - Very happy - Generally happy - Neither happy nor unhappy - Generally unhappy - Very unhappy |  |  |  |
| 185. How often did you feel in despair over the last seven days? | - Never - Occasionally - Sometimes - Often - All the time |  |  |  |

**SUBSTANCES - ASSIST-Lite**

Psychoactive substances are substances that, when used, affect a person’s mental processes, e.g., perception, consciousness, thoughts, mood and/or emotions. These substances can be smoked, swallowed, snorted, inhaled, or injected. These substances include tobacco, alcohol, cannabis, stimulants, sedatives, and opioids.

The following questions ask about psychoactive substances that you may have used in the past 3 months only.

| **Questions** | **Variables** | **6 wk pre** | **6 wk post** | **1 yr post** |
| --- | --- | --- | --- | --- |
| 186. Did you smoke a cigarette containing tobacco? | - Yes - No |  |  |  |
| 187. Did you usually smoke more than 10 cigarettes each day?   *Will only display if ‘yes’ is selected in responding to the previous question.* | - Yes - No |  |  |  |
| 188. Did you usually smoke within 30 minutes after waking?   *Will only display if ‘yes’ is selected to whether a cigarette has been smoked.* | - Yes - No |  |  |  |
| 189. Did you have a drink containing alcohol? | - Yes - No |  |  |  |
| 190. On any occasion, did you drink more than 4 standard drinks of alcohol?  *If you are unsure how many standard drinks you have had, you may like to refer to this* [*Standard Drinks Guide*](https://www.health.gov.au/topics/alcohol/about-alcohol/standard-drinks-guide)*, which will open in a separate window.*   *Will only display if ‘yes’ is selected in responding to the previous question.* | - Yes - No |  |  |  |
| 191. Have you tried and failed to control, cut down or stop drinking?   *Will only display if ‘yes’ is selected in responding to drinking any alcohol.* | - Yes - No |  |  |  |
| 192. Has anyone expressed concern about your drinking?   *Will only display if ‘yes’ is selected in responding to drinking any alcohol.* | - Yes - No |  |  |  |
| 193. Did you use cannabis? | - Yes - No |  |  |  |
| 194. Have you had a strong desire or urge to use cannabis at least once a week or more often?   *Will only display if ‘yes’ is selected in responding to the previous question.* | - Yes - No |  |  |  |
| 195. Has anyone expressed concern about your use of cannabis?   *Will only display if ‘yes’ is selected in responding to using any cannabis.* | - Yes - No |  |  |  |
| 196. Did you use an amphetamine-type stimulant, or cocaine, or a stimulant medication not as prescribed?  *Amphetamine-type stimulants include amphetamine, dexamphetamine, methamphetamine, and ecstacy (MDMA).* | - Yes - No |  |  |  |
| 197. Did you use a stimulant at least once each week or more often?   *Will only display if ‘yes’ is selected in responding to the previous question.* | - Yes - No |  |  |  |
| 198. Has anyone expressed concern about your use of a stimulant?   *Will only display if ‘yes’ is selected in responding to using any type of stimulant.* | - Yes - No |  |  |  |
| 199. Did you use a sedative or sleeping medication not as prescribed? | - Yes - No |  |  |  |
| 200. Have you had a strong desire or urge to use a sedative or sleeping medication at least once a week or more often?   *Will only display if ‘yes’ is selected in responding to the previous question.* | - Yes - No |  |  |  |
| 201. Has anyone expressed concern about your use of a sedative or sleeping medication?   *Will only display if ‘yes’ is selected in responding to use of a sedative/sleeping medication not as prescribed.* | - Yes - No |  |  |  |
| 202. Did you use a street opioid (e.g., heroin) or an opioid-containing medication not as prescribed? | - Yes - Np |  |  |  |
| 203. Have you tried and failed to control, cut down or stop using an opioid?   *Will only display if ‘yes’ is selected in responding to the previous question.* | - Yes - No |  |  |  |
| 204. Has anyone expressed concern about your use of an opioid?   *Will only display if ‘yes’ is selected in responding to using a street opioid.* | - Yes - No |  |  |  |
| 205. Did you use any other psychoactive substances in the past 3 months? If yes, please let us know what you took. | [open text] |  |  |  |
| 206. Do you use psychoactive substances to cope with chest/breast dysphoria? | - Never - Sometimes - Often - Very often - Does not apply to me |  |  |  |
| 207. Please tell us more about any of your answers above if you wish to: | [open text] |  |  |  |

# SOCIAL ACCEPTANCE AND DISCRIMINATION

We want to understand experiences of misgendering, safety in public, discrimination, and social acceptance and how these impact on mental health. Misgendering is when someone incorrectly assumes your gender. This may include using incorrectly gendered language, pronouns and/or name.

This set of questions ask about non-affirming public interactions where gender is assumed based on appearance.

| **Questions** | **Variables** | **6 wk pre** | **6 wk post** | **1 yr post** |
| --- | --- | --- | --- | --- |
| 208. How often have you been misgendered within the last 6 months? | - Never - Sometimes - Often - Very often - Prefer not to say |  |  |  |

In the past 6 months, how often have you been misgendered by each of the following:

| **Questions** | **Variables** | **6 wk pre** | **6 wk post** | **1 yr post** |
| --- | --- | --- | --- | --- |
| 209. My partner/s   *Will only display if ‘never’ or ‘prefer not to say’ are* ***not*** *selected in response to being misgendered in the past 6 months.* | - Never - Sometimes - Often - Very often - Does not apply to me - Prefer not to say |  |  |  |
| 210. My friends   *Will only display if ‘never’ or ‘prefer not to say’ are* ***not*** *selected in response to being misgendered in the past 6 months.* | - Never - Sometimes - Often - Very often - Does not apply to me - Prefer not to say |  |  |  |
| 211. My immediate family   *Will only display if ‘never’ or ‘prefer not to say’ are* ***not*** *selected in response to being misgendered in the past 6 months.* | - Never - Sometimes - Often - Very often - Does not apply to me - Prefer not to say |  |  |  |
| 212. My extended family   *Will only display if ‘never’ or ‘prefer not to say’ are* ***not*** *selected in response to being misgendered in the past 6 months.* | - Never - Sometimes - Often - Very often - Does not apply to me - Prefer not to say |  |  |  |
| 213. My colleagues   *Will only display if ‘never’ or ‘prefer not to say’ are* ***not*** *selected in response to being misgendered in the past 6 months.* | - Never - Sometimes - Often - Very often - Does not apply to me - Prefer not to say |  |  |  |
| 214. My peers   *Will only display if ‘never’ or ‘prefer not to say’ are* ***not*** *selected in response to being misgendered in the past 6 months.* | - Never - Sometimes - Often - Very often - Does not apply to me - Prefer not to say |  |  |  |
| 215. My housemate/roommate   *Will only display if ‘never’ or ‘prefer not to say’ are* ***not*** *selected in response to being misgendered in the past 6 months.* | - Never - Sometimes - Often - Very often - Does not apply to me - Prefer not to say |  |  |  |
| 216. People who don’t know me (e.g., strangers in public)   *Will only display if ‘never’ or ‘prefer not to say’ are* ***not*** *selected in response to being misgendered in the past 6 months.* | - Never - Sometimes - Often - Very often - Does not apply to me - Prefer not to say |  |  |  |
| 217. Health professionals   *Will only display if ‘never’ or ‘prefer not to say’ are* ***not*** *selected in response to being misgendered in the past 6 months.* | - Never - Sometimes - Often - Very often - Does not apply to me - Prefer not to say |  |  |  |
| 218. Auxiliary staff within health services (e.g., receptionist)   *Will only display if ‘never’ or*  *‘prefer not to say’ are* ***not***  *selected in response to being*  *misgendered in the past 6*  *months.* | - Never - Sometimes - Often - Very often - Does not apply to me - Prefer not to say |  |  |  |
| 219. People in human services (e.g. Centrelink, Medicare)   *Will only display if ‘never’ or*  *‘prefer not to say’ are* ***not***  *selected in response to being*  *misgendered in the past 6*  *months.* | - Never - Sometimes - Often - Very often - Does not apply to me - Prefer not to say |  |  |  |
| 220. How has misgendering impacted your mental health?   *Will only display if ‘never’ or ‘prefer not to say’ are* ***not*** *selected in response to being misgendered in the past 6 months.* | [open text] |  |  |  |
| 221. When you are gendered correctly, how does this impact your mental health? | [open text] |  |  |  |
| 222. In the past 6 months how often have you had any experiences of verbal abuse or harassment? | - Never - Sometimes - Often - Very often - Prefer not to say |  |  |  |
| 223. Within the last 6 months how often have you been worried about your safety in public spaces because of your gender? | - Never - Sometimes - Often - Very often - Does not apply to me - Prefer not to say |  |  |  |
| 224. Within the last 6 months how often have you been worried about your safety in public bathrooms or change rooms because of your gender? | - Never - Sometimes - Often - Very often - Does not apply to me - Prefer not to say |  |  |  |
| 225. Within the last 6 months how often have you been worried about your safety whilst on public transport because of your gender? | - Never - Sometimes - Often - Very often - Does not apply to me - Prefer not to say |  |  |  |
| 226. Within the last 6 months how often have you been worried about your safety in your current living situation because of your gender? | - Never - Sometimes - Often - Very often - Prefer not to say |  |  |  |
| 227. Within the last 6 months how often have you been worried about your safety whilst at work because of your gender? | - Never - Sometimes - Often - Very often - Does not apply to me - Prefer not to say |  |  |  |
| 228. Within the last 6 months how often have you been worried about your safety whilst studying/training/in an education setting because of your gender? | - Never - Sometimes - Often - Very often - Does not apply to me - Prefer not to say |  |  |  |
| 229. Do you have any further comments about safety in the last 6 months that you would like to share? | [open text] |  |  |  |
| 230. If you would like to, please comment on how your experience of misgendering has changed following surgery and recovery: | [open text] |  |  |  |

# CLOSING REFLECTION AND EVALUATION

These next questions ask how you feel about the results of your top surgery.

| **Questions** | **Variables** | **6 wk pre** | **6 wk post** | **1 yr post** |
| --- | --- | --- | --- | --- |
| 231. Looking back on your top surgery, are there any things you would reconsider or do differently? (Select all that apply) | - I would have chosen a different surgeon - I would have had a different surgical procedure - I wish I had surgery earlier in life - I wish I had surgery later in life - The impact on my finances - The positive impact on my relationship/s - The negative impact on my relationship/s - Taking more care during healing process (scar care, limiting movement etc.) - Having more mental health and/or physical support - I would not have had surgery at all - There is nothing I would change - Prefer not to say - Other/something else: [open text] |  |  |  |
| 232. Please tell us more about this if you want to: | [open text] |  |  |  |
| 233. How satisfied are you with your chest/breasts now that you have had surgery? | - Completely satisfied - Very satisfied - Moderately satisfied - Slightly satisfied - Not at all satisfied - Prefer not to say |  |  |  |
| 234. How does/do your chest/breasts align with your pre-surgery expectations? | - I’m happier than I expected with the results - The results are what I expected - I’m not as happy as I expected - Prefer not to say |  |  |  |
| 235. How satisfied are you with your nipples? | - Completely satisfied - Very satisfied - Moderately satisfied - Slightly satisfied - Not at all satisfied - Does not apply to me - Prefer not to say |  |  |  |
| 236. How satisfied are you with scarring? | - Completely satisfied - Very satisfied - Moderately satisfied - Slightly satisfied - Not at all satisfied - Prefer not to say |  |  |  |
| 237. How satisfied are you with contouring?  *Chest contouring refers to contouring the skin and tissue of the chest to give a traditionally masculine appearance.* | - Completely satisfied - Very satisfied - Moderately satisfied - Slightly satisfied - Not at all satisfied - Prefer not to say |  |  |  |
| 238. How satisfied are you with skin appearance? (e.g., stretch marks, puckering of skin) | - Completely satisfied - Very satisfied - Moderately satisfied - Slightly satisfied - Not at all satisfied - Prefer not to say |  |  |  |
| 239. How satisfied are you with symmetry? | - Completely satisfied - Very satisfied - Moderately satisfied - Slightly satisfied - Not at all satisfied - Prefer not to say |  |  |  |
| 240. *If applicable*, how satisfied are you with your breast size? | - Completely satisfied - Very satisfied - Moderately satisfied - Slightly satisfied - Not at all satisfied - Does not apply to me - Prefer not to say |  |  |  |
| 241. *If applicable*, how satisfied are you with the flatness of your chest? | - Completely satisfied - Very satisfied - Moderately satisfied - Slightly satisfied - Not at all satisfied - Does not apply to me - Prefer not to say |  |  |  |
| 242. What are the benefits, strengths, and positive aspects that have come from top surgery? | [open text] |  |  |  |
| 243. Is there anything else you would like to tell us about your satisfaction with the outcomes? | [open text] |  |  |  |
| 244. This is the end of the survey, is there anything else you would like to tell us? | [open text] |  |  |  |
| 245. Would you like to be informed about the results of this study? | - Yes - No |  |  |  |

Thank you for completing this questionnaire.

*If any of the questions we just asked were distressing, we urge you to contact Qlife LGBTI phone and chat counselling and info service (1800 184 527 www.qlife.org.au), Lifeline (13 11 14 www.lifeline.org.au), Suicide Call Back Service (1300 659 467* [*www.suicidecallbackservice.org.au/*](http://www.suicidecallbackservice.org.au/)*), beyondblue (1300 22 4636* [*www.beyondblue.org.au*](http://www.beyondblue.org.au)*), Kids Helpline (1800 55 1800* [*www.kidshelpline.com.au*](http://www.kidshelpline.com.au)*), 13YARN (13 92 76* [*www.13yarn.com.au*](http://www.13yarn.com.au)*), The Butterfly Foundation (1800 33 4673* [*https://butterfly.org.au/*](https://butterfly.org.au/)*) or any other support service.*
